# Data-based RNA-seq simulations by binomial thinning

**DOI:** 10.1186/s12859-020-3450-9

**Published:** 2020-05-24

**Authors:** David Gerard

**Affiliations:** Department of Mathematics and Statistics, American University, Massachusetts Ave NW, Washington, DC, 20016 USA

**Keywords:** RNA-seq, Simulation, Differential expression, Factor analysis, Confounders, Scaling factors

## Abstract

**Background:**

With the explosion in the number of methods designed to analyze bulk and single-cell RNA-seq data, there is a growing need for approaches that assess and compare these methods. The usual technique is to compare methods on data simulated according to some theoretical model. However, as real data often exhibit violations from theoretical models, this can result in unsubstantiated claims of a method’s performance.

**Results:**

Rather than generate data from a theoretical model, in this paper we develop methods to add signal to real RNA-seq datasets. Since the resulting simulated data are not generated from an unrealistic theoretical model, they exhibit realistic (annoying) attributes of real data. This lets RNA-seq methods developers assess their procedures in non-ideal (model-violating) scenarios. Our procedures may be applied to both single-cell and bulk RNA-seq. We show that our simulation method results in more realistic datasets and can alter the conclusions of a differential expression analysis study. We also demonstrate our approach by comparing various factor analysis techniques on RNA-seq datasets.

**Conclusions:**

Using data simulated from a theoretical model can substantially impact the results of a study. We developed more realistic simulation techniques for RNA-seq data. Our tools are available in the seqgendiff R package on the Comprehensive R Archive Network: https://cran.r-project.org/package=seqgendiff.

## Background

Due to its higher signal-to-noise ratio, larger range of detection, and its ability to measure a priori unknown genes, RNA-seq has surpassed microarrays as the technology of choice to measure gene expression [1]. With the advent of single-cell RNA-seq technologies, researchers now even have the ability to explore expression variation at the individual cell level [2]. This presents exciting opportunities for researchers to characterize the expression heterogeneity between and within organisms, and has brought about a plentiful flow of new datasets. In the wake of these new data, an explosion of methods has been developed to analyze them. In “Application: evaluating differential expression analysis” section, “Application: evaluating confounder adjustment” section, “Application: evaluating effects of library size heterogeneity” section, and “Application: evaluating factor analysis” section we provide a large (yet terribly incomplete) list of methods designed to analyze RNA-seq data.

The typical pipeline to evaluate a method is to first simulate data according to some theoretical model, then compare it to competing methods on these simulated data and show it to be superior in some fashion. This way of evaluation can be useful to see how a method works in ideal scenarios. However, real data rarely live in ideal scenarios. Real data often exhibit unwanted variation beyond that assumed by a model [3]. Theoretical distributional assumptions are also difficult to verify, and are sometimes mired in controversy [4].

In this paper, we propose an alternative approach. Rather than generate data with a prespecified signal according to some modeling assumptions, we take a real RNA-seq dataset and add a prespecified signal to it. The main advantage of our approach is that any unwanted variation in the real data is maintained in the simulated data, and this unwanted variation need not be prespecified by the researcher. The way we add signal does carry assumptions, but they are flexible (Supplementary Section S1.2, Additional file 1). And we believe that this way of simulation, compared to simulating under a theoretical model, allows researchers to more realistically evaluate their methods.

This manuscript essentially generalizes the simulation techniques proposed in [5, 6], and [7]. These previous papers use binomial thinning (the approach used in this paper) in the case where there are just two groups that are differentially expressed (hereafter, the “two-group model”). Binomial thinning is the process of subsampling counts using the binomial distribution. This subsampling is applied to different individuals heterogeneously to add signal to the observed counts. These papers did not develop methods for more complicated design scenarios, they did not present user-friendly software implementations for their simulation techniques, and they did not justify their simulation techniques as broadly. Here, we allow for arbitrary experimental designs, we release software for users to implement their own simulations, and we justify our techniques using very flexible assumptions.

There has been some other previous work on “data-based” simulations in expression analyses. Datasets resulting from data-based simulations (sometimes called “plasmodes” [8]) have been used in microarray studies before the development of RNA-seq [9, 10]. All RNA-seq data-based simulation methods have so far operated in the two-group (or finite-group) model, without any ability to simulate data from arbitrary experimental designs. Rocke et al. [11] and [12] randomly shuffled group indicators in the two-group model, resulting in completely null data, and methods can be evaluated on their ability to control for type I error when the data are all null. Rigaill et al. [13], in addition to generating null data by randomly shuffling group labels, incorporate multiple datasets to create some non-null genes within their simulated datasets. Benidt and Nettleton [14] use a count-swapping algorithm in the two-group model to create differentially expressed genes when one already has two treatment groups. Kvam et al. [15, 16], and [17] create non-null genes by multiplying counts for all individuals in a group by the fold-change in mean expression. [18] uses a binomial distribution approach to uniformly decrease the sequencing depth of an entire dataset (but not to add differentially expressed genes). Concerning non-data-based methods, [19] and [20] use real RNA-seq data to estimate the parameters in a data-generating model before simulating data from the theoretical model using these estimated parameter values. Our work is the first to extend data-based RNA-seq simulation beyond the finite-group model.

Our paper is organized as follows. We first list the goals and assumptions of our simulation scheme (“Goals and assumptions” section) before motivating it with four applications (“Application: evaluating differential expression analysis” section, “Application: evaluating confounder adjustment” section, “Application: evaluating effects of library size heterogeneity” section, and “Application: evaluating factor analysis” section) and describing our process of simulating RNA-seq in detail (“Generating modified RNA-seq data” section). We then demonstrate how our approach can more accurately preserve structure in a real dataset compared to simulating a dataset from a theoretical model (“Features of real data” section). We show that this can alter the conclusions of a differential expression analysis simulation study (“Effects on differential expression analysis simulations” section). We then apply our simulation approach by comparing five factor analysis methods using the GTEx data [21] (“Evaluating factor analyses” section). We finish with a discussion and conclusions (“Discussion” section and “Conclusions” section).

We adopt the following notation. We denote matrices by bold uppercase letters (***A***), vectors by bold lowercase letters (***a***), and scalars by non-bold letters (*a* or *A*). Indices typically run from 1 to their uppercase version, e.g. *a*=1,2,…,*A*. Where there is no chance for confusion, we let non-bold versions of letters represent the scalar elements of matrices and vectors. So *a*_*ij*_ is the (*i*,*j*)th element of ***A***, while *a*_*i*_ is the *i*th element of ***a***. We let ***1***_*A*_ denote the *A*-vector of 1’s and ***1***_*A*×*B*_ the *A*×*B* matrix of 1’s. The matrix transpose is denoted by $\boldsymbol {A}^{\intercal }$.

## Methods

### Goals and assumptions

We will now describe the goals and assumptions of our simulation method, which relies on a researcher having access to a real RNA-seq dataset. Suppose a researcher has a matrix $\boldsymbol {Y} \in \mathbb {R}^{G \times N}$ of RNA-seq read-counts for *G* genes and *N* individuals. Also suppose a researcher has access to a design matrix $\boldsymbol {X}_{1} \in \mathbb {R}^{N \times P_{1}}$ with *P*_1_ variables. The availability of ***X***_1_ is optional, not essential to the method, and is mostly for descriptive purposes. We assume the RNA-seq counts, ***Y***, are generated according to the following model:

1 $$ \begin{array}{ll}  y_{gn} &\sim \text{Poisson}(2^{\theta_{gn}}), \text{ and}\\ \boldsymbol{\Theta} &= \boldsymbol{\mu}\boldsymbol{1}_{N}^{\intercal} + \boldsymbol{B}_{1}\boldsymbol{X}_{1}^{\intercal} + \boldsymbol{A}\boldsymbol{Z}^{\intercal} + \boldsymbol{\Omega}, \end{array}  $$

where

- $\boldsymbol {\mu } \in \mathbb {R}^{G}$ is a vector of intercept terms for the genes,
- $\boldsymbol {B}_{1} \in \mathbb {R}^{G \times P_{1}}$ is the corresponding coefficient matrix of ***X***_1_,
- $\boldsymbol {Z} \in \mathbb {R}^{N \times K}$ is a matrix of unobserved surrogate variables,
- $\boldsymbol {A} \in \mathbb {R}^{G \times K}$ is the corresponding coefficient matrix of ***Z***, and
- $\boldsymbol {\Omega } \in \mathbb {R}^{G \times N}$ represents all other unwanted variation not accommodated by the other terms in the model,

where ***μ***, ***B***_1_, ***Z***, ***A***, and ***Ω*** are all unknown. Given the above data-generating process, suppose a user provides the following (known) elements:

- $\boldsymbol {X}_{2} \in \mathbb {R}^{N \times P_{2}}$, a design matrix with fixed rows (see note 3 below),
- $\boldsymbol {B}_{2} \in \mathbb {R}^{G \times P_{2}}$, the coefficient matrix corresponding to ***X***_2_,
- $\boldsymbol {X}_{3} \in \mathbb {R}^{N \times P_{3}}$, a design matrix with rows that can be permuted (see note 3 below), and
- $\boldsymbol {B}_{3} \in \mathbb {R}^{G \times P_{3}}$, the coefficient matrix corresponding to ***X***_3_.

Our goal is to generate a matrix $\tilde {\boldsymbol {Y}} \in \mathbb {R}^{G\times N}$ from ***Y*** such that

2 $$\begin{array}{*{20}l}\begin{aligned}  \tilde{y}_{gn} &\sim \text{Poisson}(2^{\tilde{\theta}_{gn}}), \text{ and}\\ \tilde{\boldsymbol{\Theta}} &= \tilde{\boldsymbol{\mu}}\boldsymbol{1}_{N}^{\intercal} + \boldsymbol{B}_{1}\boldsymbol{X}_{1}^{\intercal} + \boldsymbol{B}_{2}\boldsymbol{X}_{2}^{\intercal} + \boldsymbol{B}_{3}\boldsymbol{X}_{3}^{\intercal}\boldsymbol{\Pi}^{\intercal} + \boldsymbol{A}\boldsymbol{Z}^{\intercal} + \boldsymbol{\Omega}, \end{aligned} \end{array} $$

where

- $\boldsymbol {\Pi } \in \mathbb {R}^{N\times N}$ is a random permutation matrix, whose distribution controls the level of association between the columns of ***ΠX***_3_ and the columns of ***Z***, and
- $\tilde {\boldsymbol {\mu }}$ is a new vector of intercept terms for the genes.

We will provide the details on how to generate $\tilde {\boldsymbol {Y}}$ from ***Y*** in “Generating modified RNA-seq data” section. But we would like to first provide some notes below, and then discuss the applications of being able to generate (2) from (1). **Note 1:** For simplicity we use the Poisson distribution in the main text (Eqs. (1) and (2)). However, our approach is valid under much more general assumptions. In particular, we note that if the counts were generated according to a negative binomial distribution, a zero-inflated negative binomial distribution, or even a mixture of binomials and negative binomials, then our simulation scheme still preserves the structure of the data (Supplementary Section S1.2, Additional file 1). However, even when our general modeling assumptions are violated, one can show (via the law of total expectation) that if log2(*E*[***Y***])=***Θ***, then $\log _{2}(E[\tilde {\boldsymbol {Y}}]) = \tilde {\boldsymbol {\Theta }}$, where we are taking element-wise logarithms of *E*[***Y***] and $E[\tilde {\boldsymbol {Y}}]$. Thus, our procedure will produce the correct mean log2-fold change in the new dataset, but the resulting mean/variance relationship might not be as assumed. **Note 2:** The ***Ω*** term in (1) and (2) represents the realistic and annoying features of the data. In ideal situations, ***Ω***=***0***_*G*×*N*_. However, most datasets likely include non-zero ***Ω***, and so assessing a method’s ability to be robust in the presence of ***Ω***, without the researcher having to prespecify ***Ω***, is the key strength of our simulation approach. **Note 3:** As described below, we include both ***X***_2_ and ***X***_3_ in (2) to control different aspects of a simulation study. One may control the level of association between the columns of ***X***_1_ and ***X***_2_ as these are both observed and fixed by the user. The inclusion of ***X***_3_ and ***Π*** allows us to try to control the level of association between ***ΠX***_3_ and ***Z***.

Before we discuss obtaining (2) from (1), we point out four potential applications of this simulation approach: (i) evaluating differential expression analyses (“Application: evaluating differential expression analysis” section), (ii) evaluating confounder adjustment approaches (“Application: evaluating confounder adjustment” section), (iii) evaluating the effects of library size heterogeneity on differential expression analyses (“Application: evaluating effects of library size heterogeneity” section), and (iv) evaluating factor analysis methods (“Application: evaluating factor analysis” section).

### Application: evaluating differential expression analysis

One of the more common applications of RNA-seq data is estimating and testing for differences in gene expression between two groups. Many packages and techniques exist to perform this task [22–39, among others], and so developing approaches and software to compare these different software packages would be of great utility to the scientific community. Generating data from the two-group model is a special case of (1) and (2), where

3 $$\begin{array}{*{20}l} \boldsymbol{\Theta} &= \boldsymbol{\mu}\boldsymbol{1}_{N}^{\intercal} + \boldsymbol{\Omega}, \end{array} $$

4 $$\begin{array}{*{20}l} \tilde{\boldsymbol{\Theta}} &= \tilde{\boldsymbol{\mu}}\boldsymbol{1}_{N}^{\intercal} + \boldsymbol{b}\boldsymbol{x}^{\intercal}\boldsymbol{\Pi}^{\intercal} + \boldsymbol{\Omega}, \end{array} $$

and $\boldsymbol {\Pi }\boldsymbol {x} \in \mathbb {R}^{N}$ contains a single indicator variable, indicating membership to one of two groups. Researchers may specify ***b*** and ***x*** and evaluate a method’s ability to (i) estimate ***b*** and (ii) detect which genes have non-zero *b*_*g*_.

In many settings, a researcher may want to specify the distribution of the *b*_*g*_’s (the elements of ***b***). Our software implementation allows for this. In addition, following [40], we allow researchers to specify the distribution of $b_{g}/s_{g}^{\alpha }$, where *s*_*g*_ is the sample standard deviation of the *g*th row of log2(***Y***+0.5), and *α* is a user-specified constant. Allowing for *α*=0 corresponds to the scenario of specifying the distribution of the effects, while allowing for *α*=1 corresponds to specifying the *p*-value prior of [41].

Though the two-group model is perhaps the most common scenario in differential expression analysis, our method also allows for arbitrary design matrices. Such design matrices have applications in many types of expression experiments [24, 42–44], and so the ability to simulate arbitrary designs gives researchers another tool to evaluate their methods in more complicated scenarios.

### Application: evaluating confounder adjustment

Unobserved confounding / batch effects / surrogate variables / unwanted variation has been recognized as a serious impediment to scientific studies in the modern “omics” era [3]. As such, there is a large literature on accounting for unwanted variation, particularly in RNA-seq studies [5, 6, 45–72, among others]. The glut of available methods indicates a need to realistically compare these methods.

Typically, the form and strength of any unobserved confounding is not known. So one way to assess different confounder adjustment methods would be to assume model (1) and add signal to the data resulting in the following submodel of (2):

5 $$\begin{array}{*{20}l} \tilde{\boldsymbol{\Theta}} &= \tilde{\boldsymbol{\mu}}\boldsymbol{1}_{N}^{\intercal} + \boldsymbol{B}_{1}\boldsymbol{X}_{1}^{\intercal} + \boldsymbol{B}_{3}\boldsymbol{X}_{3}^{\intercal}\boldsymbol{\Pi}^{\intercal} + \boldsymbol{A}\boldsymbol{Z}^{\intercal} + \boldsymbol{\Omega}. \end{array} $$

A researcher would then explore how close a method’s estimate of ***B***_3_ is to the truth (assuming the researcher may use both ***X***_1_ and ***ΠX***_3_ to obtain this estimate). The researcher can control the correlation between the columns of ***ΠX***_3_ and the columns of ***Z*** by specifying the distribution of ***Π*** (as described in “Generating modified RNA-seq data” section). Intuitively, the stronger the correlation between the columns of ***X***_3_ and the columns of ***Z***, the more difficult the confounder adjustment problem. This approach was used in the two-group model in [5] and [6], but not for general design matrices.

### Application: evaluating effects of library size heterogeneity

“Library size” corresponds to the number of reads an individual sample contains. Adjusting for library size is surprisingly subtle and difficult, and thus many techniques have been proposed to perform this adjustment [73–77]. The most commonly-used techniques can be viewed as a form of confounder adjustment [5]. For most methods, this form of confounder adjustment corresponds to setting one column of ***A*** in (1) to be ***1***_*G*_ and estimating the corresponding column in ***Z*** using some robust method that assumes that the majority of genes are non-differentially expressed.

One way to evaluate the performance of a library size adjustment procedure is to see how effect size estimates change when the samples are thinned, changing the library size. First, assume we are operating in the following submodel of (1):

6 $$\begin{array}{*{20}l}  \boldsymbol{\Theta} = \boldsymbol{\mu}\boldsymbol{1}_{N}^{\intercal} + \boldsymbol{B}_{1}\boldsymbol{X}_{1}^{\intercal} + \boldsymbol{1}_{G}\boldsymbol{z}^{\intercal} + \boldsymbol{\Omega}. \end{array} $$

A researcher may specify (i) additional signal and (ii) a further amount of thinning on each sample by generating the following submodel of (2):

7 $$ {}\begin{aligned} \tilde{\boldsymbol{\Theta}} = \tilde{\boldsymbol{\mu}}\boldsymbol{1}_{N}^{\intercal} + \boldsymbol{B}_{1}\boldsymbol{X}_{1}^{\intercal} + \boldsymbol{B}_{3}\boldsymbol{X}_{3}^{\intercal}\boldsymbol{\Pi}^{\intercal} + \boldsymbol{1}_{G}\boldsymbol{x}_{2}^{\intercal} + \boldsymbol{1}_{G}\boldsymbol{x}_{3}^{\intercal}\boldsymbol{\Pi}^{\intercal} + \boldsymbol{1}_{G}\boldsymbol{z}^{\intercal} + \boldsymbol{\Omega}  \end{aligned}  $$

8 $$ {}\begin{aligned} = \tilde{\boldsymbol{\mu}}\boldsymbol{1}_{N}^{\intercal} + \boldsymbol{B}_{1}\boldsymbol{X}_{1}^{\intercal} + \boldsymbol{B}_{3}\boldsymbol{X}_{3}^{\intercal}\boldsymbol{\Pi}^{\intercal} + \boldsymbol{1}_{G}(\boldsymbol{z} + \boldsymbol{x}_{2} + \boldsymbol{\Pi}\boldsymbol{x}_{3})^{\intercal} + \boldsymbol{\Omega}. \end{aligned}  $$

To evaluate the effectiveness of a library size adjustment procedure, researchers may observe the effects on the estimates of ***B***_3_ under various amounts of library thinning (controlled by altering ***x***_2_ and ***x***_***3***_).

### Application: evaluating factor analysis

Factor analysis is a fundamental technique in every statistician’s arsenal. Since its creation by Spearman [78], literally hundreds of factor analysis / matrix decomposition / matrix factorization approaches have been developed, and new approaches are created each year to account for new features of new data [54, 79–96, to name a very few]. For RNA-seq, factor analysis methods have found applications in accounting for unwanted variation [63, 64], estimating cell-cycle state [97, 98], and general quality assessments [27]. Thus, techniques to realistically compare various factor analysis methods would be of great use to the scientific community. We demonstrate in this section how our simulation approaches can be used to evaluate factor analysis methods applied to RNA-seq.

We suppose that the RNA-seq read-counts follow the following submodel of (1):

9 $$\begin{array}{*{20}l}  \boldsymbol{\Theta} = \boldsymbol{\mu}\boldsymbol{1}_{N}^{\intercal} + \boldsymbol{A}\boldsymbol{Z}^{\intercal} + \boldsymbol{\Omega}. \end{array} $$

We then suppose that the researcher generates a modified dataset that follows the following submodel of (2):

10 $$\begin{array}{*{20}l}  \tilde{\boldsymbol{\Theta}} &= \tilde{\boldsymbol{\mu}}\boldsymbol{1}_{N}^{\intercal} + \boldsymbol{B}_{3}\boldsymbol{X}_{3}^{\intercal}\boldsymbol{\Pi}^{\intercal} + \boldsymbol{A}\boldsymbol{Z}^{\intercal} + \boldsymbol{\Omega}. \end{array} $$

We assume that a researcher applies a factor analysis to (10) to estimate a low-rank matrix with *K*+*P*_3_ factors. That is, the researcher fits the following model,

11 $$\begin{array}{*{20}l}  \log_{2}(E[\tilde{Y}]) = \boldsymbol{\mu}\boldsymbol{1}_{N}^{\intercal} + \boldsymbol{L}\boldsymbol{F}^{\intercal}, \end{array} $$

with factor matrix $\boldsymbol {F} \in \mathbb {R}^{N \times (K + P_{3})}$ and loading matrix $\boldsymbol {L} \in \mathbb {R}^{G\times (K + P_{3})}$, obtaining estimates $\hat {\boldsymbol {L}}$ and $\hat {\boldsymbol {F}}$. These estimates are obtained without using ***ΠX***_3_. A researcher may evaluate their factor analysis by

1. Assessing if any of the columns of $\hat {\boldsymbol {F}}$ are close to the columns of ***ΠX***_3_,
2. Assessing if any of the columns of $\hat {\boldsymbol {L}}$ are close to the columns of ***B***_3_, and
3. Assessing if the column-space of ***ΠX***_***3***_ is close to the column-space of $\hat {\boldsymbol {F}}$, which would be an important consideration in downstream regression analyses [45, e.g.].

In a factor analysis, the factors and loadings are only identifiable after imposing assumptions on their structure (such as sparsity or orthogonality). Thus, researchers may vary the structure of ***B***_3_ and ***ΠX***_3_ and observe the robustness of their factor analysis methods to violations of their structural assumptions.

### Generating modified RNA-seq data

We will now discuss the approach of obtaining (2) from (1). We will use the following well-known fact of the Poisson distribution, which may be found in many elementary probability texts:

#### **Lemma 1**

If *y*∼Poisson(*a*) and $\tilde {y}|y \sim \text {Bin}(y, b)$, then $\tilde {y} \sim \text {Poisson}(ab)$.

Generalizations of Lemma 1 to negative binomial distributions (and mixtures of negative binomial distributions) may be found in Section S1.2 of Additional file 1.

In the case when ***Π*** is drawn uniformly from the space of permutation matrices, we have the simplified procedure described in Procedure 1. The validity of Procedure 1 follows directly from the modeling assumptions in (1) and Lemma 1. Since $y_{gn}\sim \text {Poisson}(2^{\theta _{gn}})\phantom {\dot {i}\!}$ and $\tilde {y}_{gn}|y_{gn}\sim \text {Bin}(y_{gn},2^{q_{gn}})$, we have that $\tilde {y}_{gn}\sim \text {Poisson}(2^{\theta _{gn} + q_{gn}})$. If we set $\tilde {\theta }_{gn} = \theta _{gn} + q_{gn}$, then we have

12 $$ {}\begin{aligned} \tilde{\boldsymbol{\Theta}} &= \boldsymbol{\Theta} + \boldsymbol{Q}\\ \end{aligned}  $$

13 $$ {}\begin{aligned} &=(\boldsymbol{\mu}\boldsymbol{1}_{N}^{\intercal} + \boldsymbol{B}_{1}\boldsymbol{X}_{1}^{\intercal} + \boldsymbol{A}\boldsymbol{Z}^{\intercal} + \boldsymbol{\Omega}) + (\boldsymbol{B}_{2}\boldsymbol{X}_{2}^{\intercal} + \boldsymbol{B}_{3}\boldsymbol{X}_{3}^{\intercal}\boldsymbol{\Pi}^{\intercal} - \boldsymbol{e}\boldsymbol{1}_{N}^{\intercal})\\ \end{aligned}  $$

14 $$ {}\begin{aligned} &=(\boldsymbol{\mu} - \boldsymbol{e})\boldsymbol{1}_{N}^{\intercal} + \boldsymbol{B}_{1}\boldsymbol{X}_{1}^{\intercal} + \boldsymbol{B}_{2}\boldsymbol{X}_{2}^{\intercal} + \boldsymbol{B}_{3}\boldsymbol{X}_{3}^{\intercal}\boldsymbol{\Pi}^{\intercal} + \boldsymbol{A}\boldsymbol{Z}^{\intercal} + \boldsymbol{\Omega}\\ \end{aligned}  $$

15 $$ {}\begin{aligned} &=\tilde{\boldsymbol{\mu}}\boldsymbol{1}_{N}^{\intercal} + \boldsymbol{B}_{1}\boldsymbol{X}_{1}^{\intercal} + \boldsymbol{B}_{2}\boldsymbol{X}_{2}^{\intercal} + \boldsymbol{B}_{3}\boldsymbol{X}_{3}^{\intercal}\boldsymbol{\Pi}^{\intercal} + \boldsymbol{A}\boldsymbol{Z}^{\intercal} + \boldsymbol{\Omega}. \end{aligned}  $$

Equation (13) follows from the definition of ***Θ*** from (1) and the definition of ***Q*** from Step 4 of Procedure 1. Equation (15) follows by setting $\tilde {\boldsymbol {\mu }}$ to be ***μ***−***e***.


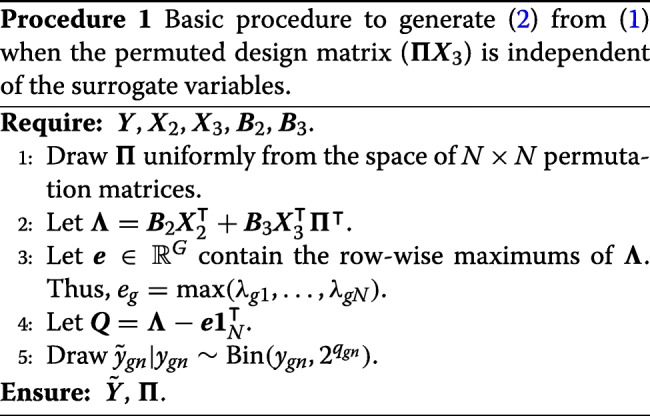


There are two main reasons to subtract the row-wise maximum from each row in Step 4 of Procedure 1: (i) this ensures that the binomial probabilities ($2^{q_{gn}}\phantom {\dot {i}\!}$) are always between 0 and 1, and (ii) this allows for minimal count-thinning while still obtaining our goal of (2). That is, the binomial probabilities will all be between 0 and 1, but they will be as close to 1 as possible while still yielding (2), thereby reducing the amount of discarded counts.

The main disadvantage to Procedure 1 is that the surrogate variables (***Z***) will be independent of the user-specified covariates (***ΠX***_3_). To allow the user to control the level of association between the surrogate variables and the user-provided variables, we propose using Procedure 2 to choose ***Π***, rather than drawing ***Π*** uniformly from the space of permutation matrices. In brief, the user specifies a “target correlation” matrix, $\boldsymbol {R} \in \mathbb {R}^{P_{3}\times K}$, where *r*_*ik*_ is what the user desires to be the correlation between the *i*th column of ***ΠX***_3_ and the *k*th column of ***Z***. We then estimate the surrogate variables either using a factor analysis (such as the truncated singular value decomposition) or surrogate variable analysis [45, 49]. Note that this estimate of ***Z*** is only used to permute the rows of ***X***_3_ and is otherwise not included in the simulated data. We then draw a new random matrix $\boldsymbol {U} \in \mathbb {R}^{N\times P_{3}}$ from a conditional normal distribution assuming that each row of ***U*** and ***Z*** is jointly normal with covariance matrix (16), thus the correlation between the columns of ***U*** and ***Z*** will be approximately ***R***. We then match the rows of ***X***_3_ with the rows of ***U*** using the pair-wise matching algorithm of [99], though our software provides other options to match pairs via either the Gale-Shapley algorithm [100] or the Hungarian algorithm [101]. This ensures that ***ΠX***_3_ is as close to ***U*** as possible. We denote the permutation matrix that matches the rows of ***X***_3_ with the rows of ***U*** by ***Π***.


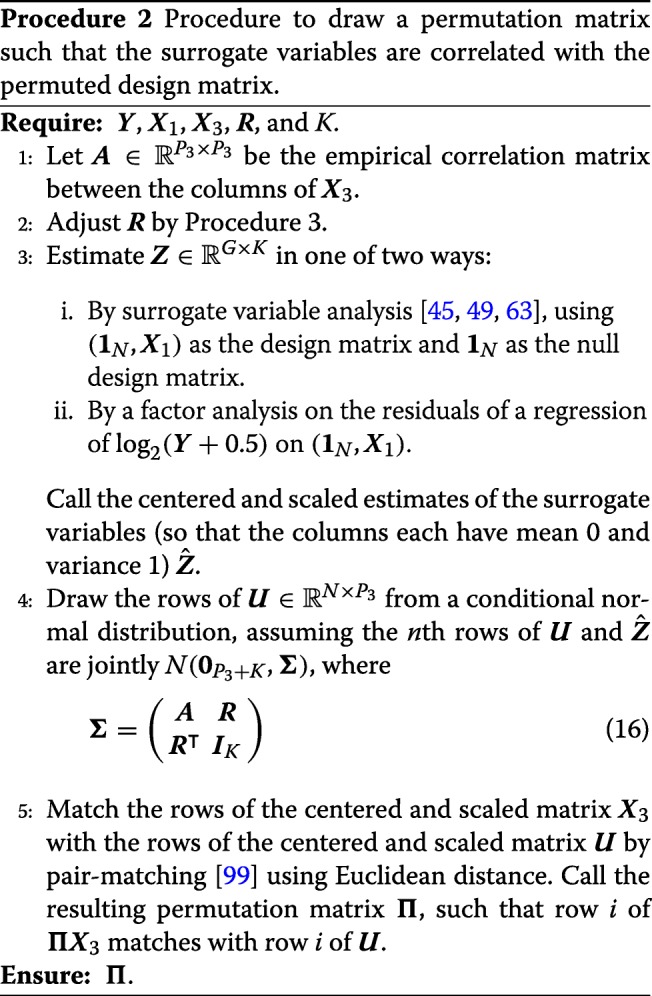



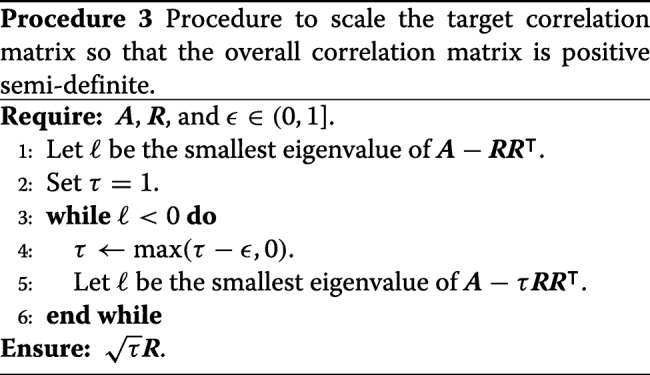


The resulting covariance matrix (16) used in Procedure 2 is not guaranteed to be positive semi-definite. Rather than demand the user specify an appropriate target correlation matrix (which might be in general difficult for the typical user), we modify the target correlation matrix using Procedure 3 to iteratively shrink ***R*** until the Schur complement condition for positive semi-definiteness [102] is satisfied.

Procedure 2 is a compromise between letting the user specify the full design matrix ***X***_3_ and letting the user specify the correlation between the columns of ***ΠX***_3_ and ***Z***. A user might want to specify the correlation between ***ΠX***_3_ and ***Z*** to evaluate factor analyses in the presence of correlated factors (“Application: evaluating factor analysis” section), or to evaluate how well confounder adjustment approaches cope in the presence of correlated confounders (“Application: evaluating confounder adjustment” section). In the simple case when ***X***_3_ and $\hat {\boldsymbol {Z}}$ are drawn from a normal distribution, Procedure 2 will permute the rows of ***X***_3_ so that ***ΠX***_3_ and $\hat {\boldsymbol {Z}}$ consistently has the correct correlation structure (Theorem S1 in Additional file 1). However, for general design matrices this will not be the case. Procedure 4 (implemented in our software) provides a Monte Carlo algorithm to estimate the true correlation given the target correlation. Basically, the estimator approximates the expected value (conditional on $\hat {\boldsymbol {Z}}$) of the Pearson correlations between the columns of ***ΠX***_3_ and the columns of $\hat {\boldsymbol {Z}}$. We justify this in an intuitive way by the law of total expectation. Consider ***x*** a single column of ***ΠX***_3_ with empirical mean and standard deviation of $\bar {x}$ and *s*_*x*_. Similarly consider ***z*** a single column of $\hat {\boldsymbol {Z}}$ with empirical mean and standard deviation of $\bar {z}$ and *s*_*z*_. Then

17 $$\begin{array}{*{20}l}  \text{cor}(x_{n}, z_{n}) &\approx E\left[\sum_{n = 1}^{N} \frac{(x_{n} - \bar{x})(z_{n} - \bar{z})}{s_{x}s_{z}}\right] \\ &= E\left[E\left[\sum_{n = 1}^{N} \frac{(x_{n} - \bar{x})(z_{n} - \bar{z})}{s_{x}s_{z}}|\boldsymbol{z}\right]\right]. \end{array} $$

The estimator in Procedure 4 is a Monte Carlo approximation to the internal expectation in (17). We explore this correlation estimator through simulation in Supplementary Section S2.1, Additional file 1.


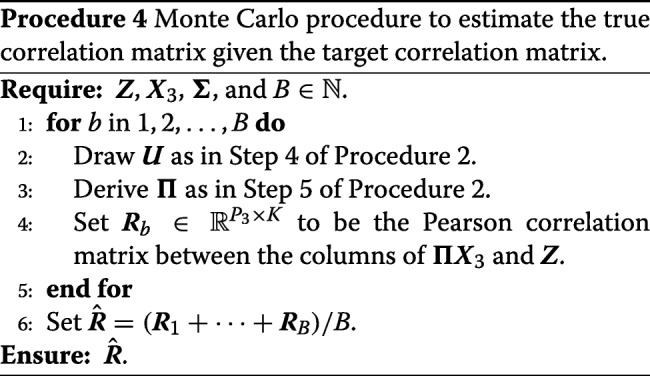


All simulation methods introduced in this paper are implemented in the seqgendiff R package, available on the Comprehensive R Archive Network: https://cran.r-project.org/package=seqgendiff.

## Results

### Features of real data

Real data exhibit characteristics that are difficult to capture by simulations. In this section, we demonstrate how our binomial thinning approach maintains these features, while simulating from a theoretical model results in unrealistic simulated RNA-seq data.

We took the GTEx muscle data [21], and filtered out all genes with a mean read-depth of less than 10 reads. This resulted in a dataset containing 18,204 genes and 564 individuals. We then randomly assigned half of the individuals to one group and half to the other group, and used our seqgendiff software to add a *N*(0,0.8^2^) log2-fold-change between groups to 25% of the genes. We similarly used the powsimR software [19] to generate data according to a theoretical negative binomial model (with parameters estimated from the GTEx muscle data), again by adding a *N*(0,0.8^2^) log2-fold-change between the two groups in 25% of the genes. The parameters estimated and used by powsimR include the mean normalized read counts per gene, the estimated library size factor per sample, and a nonparametric estimate of the mean/dispersion relationship of the counts. powsimR uses the mean normalized read counts, the estimated size factors, and the user-provided log2-fold changes to provide a mean for the negative binomial distribution. Based on this mean, it uses the estimated mean/dispersion relationship to provide a dispersion parameter for the negative binomial distribution.

The results below are from one simulation, but the results are robust and consistent across many datasets. The reader is encouraged to change the random seed in our code to explore the robustness of our conclusions.

**Fig. 1:**
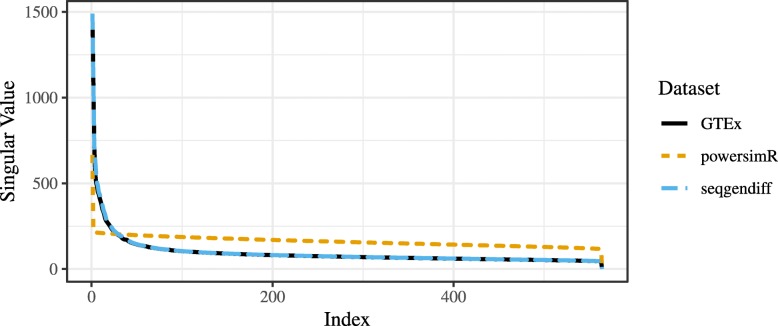
Scree Plots. Scree plots for the GTEx dataset (black), powsimR dataset (orange), and the seqgendiff dataset (blue). The singular values for the GTEx and seqgendiff datasets are almost identical

**Fig. 2:**
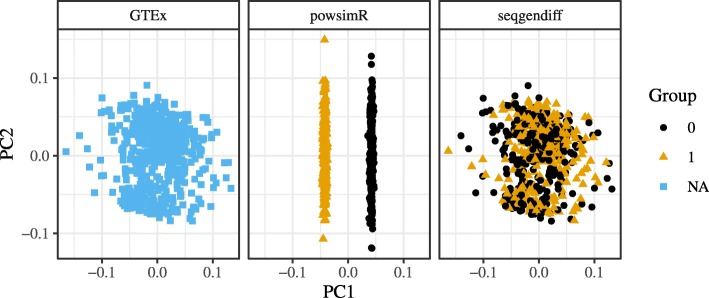
Principal Component Plots. First and second principle components for the GTEx dataset (left), the powsimR dataset (center), and the seqgendiff dataset (right). The first and second principle components of the powsimR dataset are very different from those of the GTEx and seqgendiff datasets

**Fig. 3:**
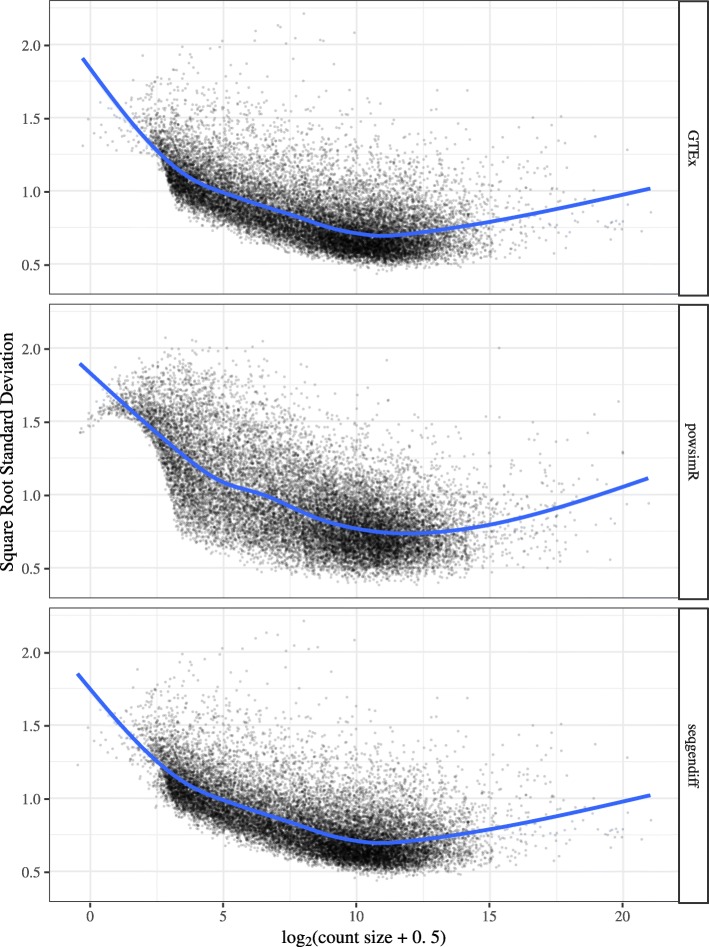
Voom plots. Voom plots [26] visualizing the mean-variance trend in RNA-seq datasets. The voom plots are visually similar for the GTEx and seqgendiff datasets. The powsimR dataset has an uncharacteristic hook near the low counts in its voom plot

The structure of the powsimR dataset is very different from that observed in the seqgendiff and GTEx datasets. There seems to be more zeros in the powsimR dataset than in the seqgendiff and GTEx datasets (Supplementary Figure S2, Additional file 1), even though we simulated the powsimR dataset under the negative binomial setting and not the zero-inflated negative binomial setting. Scree plots of the three datasets show that there are a lot more small factors influencing variation in the seqgendiff and GTEx datasets than in the powsimR dataset (Fig. 1). The main source of variation in the powsimR dataset comes from the group membership, while other (unwanted) effects dominate the variation in the seqgendiff dataset (Fig. 2). It is only the fourth principle component in the seqgendiff dataset that seems to capture the group membership (Supplementary Figure S3, Additional file 1). Though this unwanted variation exists, with such a large sample size voom-limma [26] can accurately estimate the effects (Supplementary Figure S4, Additional file 1). The voom plots (visualizing the mean-variance trend [26]) are about the same in the GTEx and seqgendiff data, but the distribution of the square-root standard deviations appears more symmetric in the powsimR dataset (Fig. 3). There is also an uncharacteristic hook in the mean-variance trend in the powsimR dataset for low-counts. These visualizations indicate that seqgendiff can generate more realistic datasets for RNA-seq simulation.

### Effects on differential expression analysis simulations

The differences in real versus simulated data (as discussed in “Features of real data” section) have real implications when evaluating methods in simulation studies. To demonstrate this, we used the GTEx muscle data to simulate RNA-seq data from the two-group model as in “Features of real data” section. We did this for *N*=10 individuals, *G*= 10,000 genes, setting 90% of the genes to be null, and generating the log2-fold change from a *N*(0,0.8^2^) distribution for the non-null genes. We simulated 500 datasets this way using both seqgendiff and powsimR. Each replication, we applied DESeq2 [27], edgeR [103], and voom-limma [26] to the simulated datasets. We evaluated the methods based on (i) false discovery proportion when using Benjamini-Hochberg [104] to control false discovery rate at the 0.05 level, (ii) power to detect non-null effects based on a 0.05 false discovery rate control threshold, and (iii) mean squared error of the estimates.

We wanted to make sure that the datasets generated from powsimR and seqgendiff were comparable, so we measured the proportion of variance explained (PVE) by the group membership for each gene, which we define as

18 $$\begin{array}{*{20}l}  V(\boldsymbol{\Pi}\boldsymbol{x}_{3}b_{3g}) / V(\log_{2}(\tilde{\boldsymbol{y}}_{g} + 0.5)), \end{array} $$

where *b*_3*g*_ is log2-fold change for gene *g*, $\tilde {\boldsymbol {y}}_{g}\in \mathbb {R}^{N}$ is the *g*th row of $\tilde {\boldsymbol {Y}}$, and *V*(·) returns the empirical variance of a vector. When we looked at the median (over the non-null genes) PVE across the datasets, the seqgendiff datasets and powsimR datasets had the same median PVE on average, though there was higher variability in the median PVE among the seqgendiff datasets (Supplementary Figure S5, Additional file 1).

**Fig. 4:**
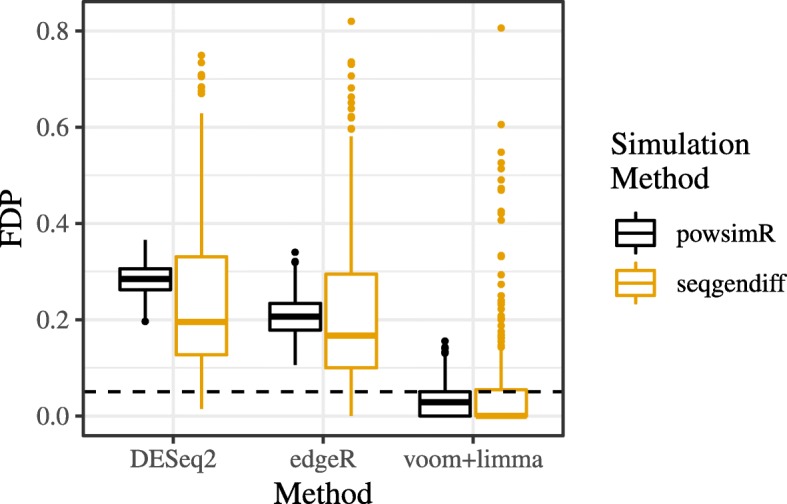
False discovery proportion of various methods. Boxplots of false discovery proportion (FDP) (*y*-axis) for various differential expression analysis methods (*x*-axis) when applied on different simulated datasets (color). Benjamini-Hochberg was used to control for false discovery rate at the 0.05 level (horizontal dashed line). Only voom-limma controls false discovery rate at the nominal level. The FDP is more variable among the seqgendiff datasets than among the powsimR datasets

Boxplots of the false discovery proportion for each method in each dataset can be found in Figure 4. Both the powsimR and seqgendiff datasets indicate that only voom-limma can control false discovery rate adequately at the nominal level. However, the results based on the seqgendiff datasets indicate that there is a lot more variability in false discovery proportion than indicated by the powsimR datasets. In particular, it does not seem uncommon for seqgendiff to generate datasets with false discovery proportions well above the nominal rate. If a researcher were using only the theoretical datasets generated by powsimR, they would be overly confident in the methods’ abilities to control false discovery proportion. Supplementary Figure S6 of Additional file 1 also indicates that methods generally have much more variable power between the seqgendiff datasets than between the powsimR datasets. Interestingly, the seqgendiff datasets indicate that methods tend to have smaller mean squared error than indicated by the powsimR datasets (Supplementary Figure S7, Additional file 1).

In Additional file 1, we also compared our simulation method to SimSeq [14] when evaluating differential expression analysis methods. We used the GTEx data [21] for both SimSeq and seqgendiff. SimSeq does not allow researchers to control the effect sizes of simulated non-null genes, as it depends on the presence of an available indicator variable that already exhibits differential expression in a real dataset. So we adjusted the effect sizes produced by seqgendiff to match those present in the GTEx data, and we found that the two data-based simulation methods behave similarly (Supplementary Figure S16, Additional file 1). It bodes well that seqgendiff produces similar results to other data-based approaches. The advantages, then, of seqgendiff over SimSeq are

1. seqgendiff can use effect sizes different than those that are already present in the observed indicator variable, while SimSeq cannot.
2. Because the effect sizes are unknown in the available indicator variable, SimSeq is unable to evaluate the estimation accuracy of effect sizes. seqgendiff can evaluate estimation accuracy.
3. seqgendiff can use more complicated designs than the finite-group model. SimSeq is limited to the finite-group model.
4. SimSeq cannot guarantee that genes that are intended to be differentially expressed in a simulated dataset are indeed differentially expressed. This depends on the quality of the available indicator variable.
5. As a minor advantage, seqgendiff is also much faster than SimSeq. On a 2.6 GHz quad-core PC running Linux with 32 GB of memory, seqgendiff took an average of 0.2 seconds to simulate a dataset, while SimSeq took an average of 51.1 seconds to simulate a dataset. A boxplot of simulation times is presented in Supplementary Figure S17 of Additional file 1.

black

### Evaluating factor analyses

As we hope we have made clear, there are many approaches to differential expression analysis (“Application: evaluating differential expression analysis” section), confounder adjustment (“Application: evaluating confounder adjustment” section), library size adjustment (“Application: evaluating effects of library size heterogeneity” section), and factor analysis (“Application: evaluating factor analysis” section). We believe it to be beyond the scope of this work to exhaustively evaluate all of these methods — especially since new methods are being developed each year. Rather, we hope our simulation procedures will be used by the research community to more realistically evaluate and benchmark their approaches to RNA-seq data analysis.

However, as a final highlight to the utility of our simulation approaches, we demonstrate these simulation techniques in one application: evaluating factor analysis methods in RNA-seq (“Application: evaluating factor analysis” section). We have chosen to highlight this particular application because it uses the more general simulation techniques beyond the two-group model, which were first demonstrated in [5].

We chose to focus on the following methods based on (i) previous use in expression studies, (ii) software availability, (iii) popularity, and (iv) ease of use.

1. Principle component analysis (PCA) [79],
2. Sparse singular value decomposition (SSVD) [93],
3. Independent component analysis (ICA) [84],
4. Factors and loadings by adaptive shrinkage (*flash*), an empirical Bayes matrix factorization approach proposed in [96], and
5. Probabilistic estimation of expression residuals (PEER) [54], a Bayesian factor analysis used in the popular PEER software to adjust for hidden confounders in gene expression studies.

All factor analysis methods were applied to the log2-counts after adding half a pseudo-count. To simulate RNA-seq data, we took the muscle GTEx data [21] and removed all genes with less than an average of 10 reads per sample. Each replicate, we added a rank-1 term. That is we assumed model (9) for the muscle GTEx data, then generated RNA-seq data such that

19 $$\begin{array}{*{20}l} \tilde{\boldsymbol{\Theta}} = \boldsymbol{\mu}\boldsymbol{1}_{N}^{\intercal} + \boldsymbol{b}_{3}\boldsymbol{x}_{3}^{\intercal}\boldsymbol{\Pi}^{\intercal} + \boldsymbol{A}\boldsymbol{Z}^{\intercal} + \boldsymbol{\Omega}, \end{array} $$

where we simulated the components of ***x***_3_ and the non-zero components of ***b***_3_ from independent normal distributions. We varied the following parameters of the simulation study:

1. The sample size: *N*∈{10,20,40}
2. The signal strength: the standard deviation of the loadings (the *b*_3*g*_’s) was set to one of {0.4,0.8}, with higher standard deviations corresponding to higher signal. These values were chosen to have the median PVE vary greatly between the two settings (Supplementary Figure S8, Additional file 1),
3. The sparsity: the proportion of loadings (the *b*_3*g*_’s) that are 0 was set to one of {0,0.9}, and
4. The target correlations of the added factor with the first unobserved factor: *r*∈{0,0.5}.

This resulted in 24 unique simulation parameter settings. We also used 1000 genes each replication. For each setting, we ran 100 replications of generating data from model (19), and fitting the factors with the five methods under study assuming model (11) after we estimated the number of hidden factors using parallel analysis [105].

We chose three metrics to evaluate the performance of the different factor analysis methods:

1. The minimum mean squared error between ***Πx***_3_ and the columns of $\hat {\boldsymbol {F}}$. To account for scale and sign unidentifiability, the estimated factors and the added factor were all scaled to have an *ℓ*^2^-norm of 1 prior to calculating the mean squared error. This measure is meant to evaluate if any of the estimated factors corresponds to the added factor.
2. The minimum mean squared error between ***b***_3_ and the columns of $\hat {\boldsymbol {L}}$. We again accounted for scale and sign unidentifiability by calculating the mean squared error after scaling the estimated and true loadings to have an *ℓ*^2^-norm of 1.
3. The angle between ***Πx***_3_ and its projection onto the column space of $\hat {\boldsymbol {F}}$. This measure is meant to evaluate if the estimated factor matrix includes ***Πx***_3_ among its unidentified factors.

The results are presented in Supplementary Figures S9–S14 of Additional file 1. Based on these figures, we have the following conclusions:

1. PEER performs very poorly when either the sparsity is high or when there are few samples. It also performs less well when the factors are correlated. A possible explanation is that PEER assumes a normal distribution on the factors and loadings, which is violated in the high-sparsity regime and is observed in the low-sparsity regime. Though, this does not explain its poor performance in small sample size settings.
2. SSVD estimates the loadings very poorly in low-sparsity regimes. This is to be as expected as SSVD assumes sparsity on the loadings. Surprisingly, though, it outperforms PCA in high sparsity regimes only when both the sample size and signal are also large.
3. ICA performs very poorly in low sparsity regimes. This is to be as expected as the normal distributions placed on the factors and loadings are a worst-case scenario for ICA. However, there is no scenario where ICA performs significantly better than PCA.
4. *flash* performs adequately in all scenarios and performs best in high-sparsity and high-signal regimes.
5. PCA performs adequately in most scenarios, and is only truly outperformed in high sparsity high signal regimes.

Based on these initial explorations, we would recommend users not use PEER, SSVD, or ICA and instead try either PCA or *flash*.

In Section S2.2 of Additional file 1, we evaluate the above factor analyses using a single cell dataset from 10X Genomics [106]. The results indicate that PCA, SSVD, and *flash* perform comparably in all simulation settings, while PEER and ICA have worse performance in some simulation settings. Though the results were less clear than when using the GTEx data.

## Discussion

We have focused on a log-linear model because of the large number of applications this generates (“Application: evaluating differential expression analysis” section, “Application: evaluating confounder adjustment” section, “Application: evaluating effects of library size heterogeneity” section, and “Application: evaluating factor analysis” section). This linearity (on the log-link scale) is represented by the structure of the ***Q*** matrix in Procedure 1. However, it is possible to replace ***Q*** by any arbitrary *G*×*N* matrix that has non-positive entries. This might be useful for simulations that study adjusting for non-linear effects, such as bias due to GC content [107]. This also might be useful for evaluating non-linear dimensionality reduction techniques such as UMAP [108] and t-SNE [109], as this allows you to introduce non-linear effects into an RNA-seq dataset. However, these non-linear effects would still be present only on the log-scale.

Our simulation procedures may be applicable beyond evaluating competing methods. Vieth et al. [19] used their simulation software to estimate power given the sample size in a differential expression analysis, and thus to develop sample size suggestions. Our simulation methods may be used similarly. Given a large RNA-seq dataset (such as the GTEx data used in this paper), one can repeatedly down-sample the number of individuals in the dataset and explore how sample size affects the power of a differential expression analysis.

Similarly, [18] already demonstrated that binomial thinning may be used for sequencing depth suggestions. That is, a researcher may repeatedly thin the libraries of the samples in a large RNA-seq dataset and explore the effects on power, thereby providing sequencing depth suggestions. Unlike [18], which does this subsampling uniformly over all counts, we allow researchers to explore the effects of heterogeneous subsampling (as in “Application: evaluating effects of library size heterogeneity” section). This might be useful if, say, researchers have more individuals in one group than in another and so wish to explore if they can sequence the larger group to a lower depth without affecting power.

In this manuscript, we have discussed our simulation techniques in the context of RNA-seq. However, our techniques would also be applicable to the comparative analysis of metagenomics methods [110]. Instead of quantifying gene expression, metagenomics quantifies gene abundances within metagenomes. Our simulation techniques could be applied in this context by taking a real metagenomics dataset and adding signal to it by binomial thinning.

## Conclusions

We developed a procedure to add a known amount of signal to any real RNA-seq dataset. We only assume that this signal comes in the form of a generalized linear model with a log-link function from a very flexible distribution. We demonstrated how real data contain features that are not captured by simulated data, and that this can cause important differences in the results of a simulation study. We highlighted our simulation approach by comparing a few popular factor analysis methods. We found that PCA and *flash* had the most robust performances across a wide range of simulation settings.

## Supplementary information

**Additional file 1** This PDF file contains theoretical considerations, simulation summaries and figures, and additional simulation details.

## Abbreviations

DESeq2: Differential expression analysis for sequence count data 2
edgeR: Empirical analysis of digital gene expression in R
FDP: False discovery proportion
*flash*: Factors and loadings by adaptive shrinkage
GTEx: Genotype-tissue expression
ICA: Independent component analysis
limma: Linear models for microarray and RNA-seq data
PCA: Principle component analysis
PEER: Probabilistic estimation of expression residuals
powsimR: Power simulations for RNA-sequencing in R
PVE: Proportion of variance explained
RNA-seq: Ribonucleic acid sequencing
seqgendiff: Sequence generation for differential expression analysis and beyond
SimSeq: Nonparametric Simulation of RNA-Seq Data
SSVD: Sparse singular value decomposition
t-SNE: t-Distributed Stochastic Neighbor Embedding
UMAP: Uniform Manifold Approximation and Projection
voom: variance modeling at the observational level

## References

[1] Wang Z, Gerstein M, Snyder M (2009). RNA-Seq: a revolutionary tool for transcriptomics. Nat Rev Genet.

[2] Hwang B, Lee JH, Bang D (2018). Single-cell RNA sequencing technologies and bioinformatics pipelines. Exp Mol Med.

[3] Leek JT, Scharpf RB, Bravo HC, Simcha D, Langmead B, Johnson WE (2010). Tackling the widespread and critical impact of batch effects in high-throughput data. Nat Rev Genet.

[4] Svensson V (2020). Droplet scRNA-seq is not zero-inflated. Nat Biotechnol.

[5] Gerard D, Stephens M. Unifying and Generalizing Methods for Removing Unwanted Variation Based on Negative Controls. Statistica Sinica. 2019;: in press. 10.5705/ss.202018.0345. 10.5705/ss.202018.0345 PMC10751021 38148787

[6] Gerard D, Stephens M. Empirical Bayes shrinkage and false discovery rate estimation, allowing for unwanted variation. Biostatistics. 2018. 10.1093/biostatistics/kxy029. 10.1093/biostatistics/kxy029 PMC8204175 29985984

[7] Lu M. Generalized Adaptive Shrinkage Methods and Applications in Genomics Studies. ProQuest Dissertations and Theses. 2018; 1:129. http://proxyau.wrlc.org/login?url=https://search.proquest.com/docview/2161785175?accountid=8285.

[8] Mehta T, Tanik M, Allison DB (2004). Towards sound epistemological foundations of statistical methods for high-dimensional biology. Nat Genet.

[9] Nettleton D, Recknor J, Reecy JM (2007). Identification of differentially expressed gene categories in microarray studies using nonparametric multivariate analysis. Bioinformatics.

[10] Gadbury GL, Xiang Q, Yang L, Barnes S, Page GP, Allison DB (2008). Evaluating Statistical Methods Using Plasmode Data Sets in the Age of Massive Public Databases: An Illustration Using False Discovery Rates. PLoS Genet.

[11] Rocke DM, Ruan L, Zhang Y, Gossett JJ, Durbin-Johnson B, Aviran S. Excess False Positive Rates in Methods for Differential Gene Expression Analysis using RNA-Seq Data. bioRxiv. 2015. Cold Spring Harbor Laboratory. 10.1101/020784. https://www.biorxiv.org/content/early/2015/06/11/020784.

[12] Sun L, Stephens M. Solving the Empirical Bayes Normal Means Problem with Correlated Noise. arXiv preprint arXiv:181207488. 2018. https://arxiv.org/abs/1812.07488.

[13] Rigaill G, Balzergue S, Brunaud V, Blondet E, Rau A, Rogier O (2016). Synthetic data sets for the identification of key ingredients for RNA-seq differential analysis. Brief Bioinformatics.

[14] Benidt S, Nettleton D (2015). SimSeq: a nonparametric approach to simulation of RNA-sequence datasets. Bioinformatics.

[15] Kvam VM, Liu P, Si Y (2012). A comparison of statistical methods for detecting differentially expressed genes from RNA-seq data. Am J Bot.

[16] Reeb P, Steibel J (2013). Evaluating statistical analysis models for RNA sequencing experiments. Front Genet.

[17] van de Wiel MA, Neerincx M, Buffart TE, Sie D, Verheul HM (2014). ShrinkBayes: a versatile R-package for analysis of count-based sequencing data in complex study designs. BMC Bioinformatics.

[18] Robinson DG, Storey JD (2014). subSeq: Determining Appropriate Sequencing Depth Through Efficient Read Subsampling. Bioinformatics.

[19] Vieth B, Ziegenhain C, Parekh S, Enard W, Hellmann I (2017). powsimR: power analysis for bulk and single cell RNA-seq experiments. Bioinformatics.

[20] Zappia L, Phipson B, Oshlack A (2017). Splatter: simulation of single-cell RNA sequencing data. Genome Biol.

[21] GTEx Consortium (2017). Genetic effects on gene expression across human tissues. Nature.

[22] Robinson MD, Smyth GK (2007). Small-sample estimation of negative binomial dispersion, with applications to SAGE data. Biostatistics.

[23] Hardcastle TJ, Kelly KA (2010). baySeq: Empirical Bayesian methods for identifying differential expression in sequence count data. BMC Bioinformatics.

[24] Van De Wiel MA, Leday GGR, Pardo L, Rue H, Van Der Vaart AW, Van Wieringen WN (2012). Bayesian analysis of RNA sequencing data by estimating multiple shrinkage priors. Biostatistics.

[25] Kharchenko PV, Silberstein L, Scadden DT, Bayesian approach to single-cell differential expression analysis (2014). Nat Methods.

[26] Law CW, Chen Y, Shi W, Smyth GK (2014). Voom: precision weights unlock linear model analysis tools for RNA-seq read counts. Genome Biol.

[27] Love MI, Huber W, Anders S (2014). Moderated estimation of fold change and dispersion for RNA-seq data with DESeq2. Genome Biol.

[28] Finak G, McDavid A, Yajima M, Deng J, Gersuk V, Shalek AK (2015). MAST: a flexible statistical framework for assessing transcriptional changes and characterizing heterogeneity in single-cell RNA sequencing data. Genome Biol.

[29] Guo M, Wang H, Potter SS, Whitsett JA, Xu Y (2015). SINCERA: A Pipeline for Single-Cell RNA-Seq Profiling Analysis. PLoS Comput Biol.

[30] Nabavi S, Schmolze D, Maitituoheti M, Malladi S, Beck AH (2015). EMDomics: a robust and powerful method for the identification of genes differentially expressed between heterogeneous classes. Bioinformatics.

[31] Delmans M, Hemberg M (2016). Discrete distributional differential expression (D3E) - a tool for gene expression analysis of single-cell RNA-seq data. BMC Bioinformatics.

[32] Korthauer KD, Chu LF, Newton MA, Li Y, Thomson J, Stewart R (2016). A statistical approach for identifying differential distributions in single-cell RNA-seq experiments. Genome Biol.

[33] Costa-Silva J, Domingues D, Lopes FM (2017). RNA-Seq differential expression analysis: An extended review and a software tool. PLoS ONE.

[34] Qiu X, Hill A, Packer J, Lin D, Ma YA, Trapnell C (2017). Single-cell mRNA quantification and differential analysis with Census. Nat Methods.

[35] Miao Z, Deng K, Wang X, Zhang X (2018). DEsingle for detecting three types of differential expression in single-cell RNA-seq data. Bioinformatics.

[36] Risso D, Perraudeau F, Gribkova S, Dudoit S, Vert JP (2018). A general and flexible method for signal extraction from single-cell RNA-seq data. Nat Commun.

[37] Van den Berge K, Perraudeau F, Soneson C, Love MI, Risso D, Vert JP (2018). Observation weights unlock bulk RNA-seq tools for zero inflation and single-cell applications. Genome Biol.

[38] Wang T, Nabavi S (2018). SigEMD: A powerful method for differential gene expression analysis in single-cell RNA sequencing data. Methods.

[39] Wang T, Li B, Nelson CE, Nabavi S (2019). Comparative analysis of differential gene expression analysis tools for single-cell RNA sequencing data. BMC Bioinformatics.

[40] Stephens M (2016). False discovery rates: a new deal. Biostatistics.

[41] Wakefield J (2009). Bayes factors for genome-wide association studies: comparison with *p*-values. Genet Epidemiol.

[42] Smyth GK (2004). Linear models and empirical Bayes methods for assessing differential expression in microarray experiments. Stat Appl Genet Mol Biol.

[43] McCarthy DJ, Chen Y, Smyth GK (2012). Differential expression analysis of multifactor RNA-Seq experiments with respect to biological variation. Nucleic Acids Res.

[44] Tang M, Sun J, Shimizu K, Kadota K (2015). Evaluation of methods for differential expression analysis on multi-group RNA-seq count data. BMC Bioinformatics.

[45] Leek JT, Storey JD (2007). Capturing heterogeneity in gene expression studies by surrogate variable analysis. PLoS Genet.

[46] Carvalho CM, Chang J, Lucas JE, Nevins JR, Wang Q, West M (2008). High-Dimensional Sparse Factor Modeling: Applications in Gene Expression Genomics. J Am Stat Assoc.

[47] Kang HM, Ye C, Eskin E (2008). Accurate discovery of expression quantitative trait loci under confounding from spurious and genuine regulatory hotspots. Genetics.

[48] Kang HM, Zaitlen NA, Wade CM, Kirby A, Heckerman D, Daly MJ (2008). Efficient control of population structure in model organism association mapping. Genetics.

[49] Leek JT, Storey JD (2008). A general framework for multiple testing dependence. Proc Natl Acad Sci.

[50] Stegle O, Kannan A, Durbin R, Winn J, Vingron M, Wong L (2008). Accounting for Non-genetic Factors Improves the Power of eQTL Studies. Research in Computational Molecular Biology: 12th Annual International Conference, RECOMB 2008, Singapore, March 30 - April 2, 2008.

[51] Friguet C, Kloareg M, Causeur D (2009). A factor model approach to multiple testing under dependence. J Am Stat Assoc.

[52] Kang HM, Sul JH, Service SK, Zaitlen NA, Kong Sy, Freimer NB (2010). Variance component model to account for sample structure in genome-wide association studies. Nat Genet.

[53] Listgarten J, Kadie C, Schadt EE, Heckerman D (2010). Correction for hidden confounders in the genetic analysis of gene expression. Proc Natl Acad Sci.

[54] Stegle O, Parts L, Durbin R, Winn J (2010). A Bayesian Framework to Account for Complex Non-Genetic Factors in Gene Expression Levels Greatly Increases Power in eQTL Studies. PLoS Comput Biol.

[55] Wu Z, Aryee MJ (2010). Subset quantile normalization using negative control features. J Comput Biol.

[56] Fusi N, Stegle O, Lawrence ND (2012). Joint Modelling of Confounding Factors and Prominent Genetic Regulators Provides Increased Accuracy in Genetical Genomics Studies. PLoS Comput Biol.

[57] Gagnon-Bartsch JA, Speed TP (2012). Using control genes to correct for unwanted variation in microarray data. Biostatistics.

[58] Stegle O, Parts L, Piipari M, Winn J, Durbin R (2012). Using probabilistic estimation of expression residuals (PEER) to obtain increased power and interpretability of gene expression analyses. Nat Protocol.

[59] Sun Y, Zhang NR, Owen AB (2012). Multiple hypothesis testing adjusted for latent variables, with an application to the AGEMAP gene expression data. Ann Appl Stat.

[60] Gagnon-Bartsch J, Jacob L, Speed T (2013). Removing Unwanted Variation from High Dimensional Data with Negative Controls. Technical Report 820.

[61] Mostafavi S, Battle A, Zhu X, Urban AE, Levinson D, Montgomery SB (2013). Normalizing RNA-sequencing data by modeling hidden covariates with prior knowledge. PLoS ONE.

[62] Yang C, Wang L, Zhang S, Zhao H (2013). Accounting for non-genetic factors by low-rank representation and sparse regression for eQTL mapping. Bioinformatics.

[63] Leek Jeffrey T. (2014). svaseq: removing batch effects and other unwanted noise from sequencing data. Nucleic Acids Research.

[64] Risso D, Ngai J, Speed TP, Dudoit S (2014). Normalization of RNA-seq data using factor analysis of control genes or samples. Nat Biotechnol.

[65] Perry PO, Pillai NS. Degrees of freedom for combining regression with factor analysis. arXiv preprint arXiv:13107269. 2015. https://arxiv.org/abs/1310.7269.

[66] Chen M, Zhou X (2017). Controlling for confounding effects in single cell RNA sequencing studies using both control and target genes. Sci Rep.

[67] Lee S, Sun W, Wright FA, Zou F (2017). An improved and explicit surrogate variable analysis procedure by coefficient adjustment. Biometrika.

[68] Wang J, Zhao Q, Hastie T, Owen AB (2017). Confounder adjustment in multiple hypothesis testing. Ann Statist.

[69] Caye K, Jumentier B, François O. LFMM 2.0: Latent factor models for confounder adjustment in genome and epigenome-wide association studies. bioRxiv. 2018.

[70] Hung Hung (2019). A robust removing unwanted variation–testing procedure via γ‐divergence. Biometrics.

[71] McKennan C, Nicolae D (2019). Accounting for unobserved covariates with varying degrees of estimability in high-dimensional biological data. Biometrika.

[72] McKennan C, Nicolae D. Estimating and accounting for unobserved covariates in high dimensional correlated data. arXiv preprint arXiv:180805895. 2018. https://arxiv.org/abs/1808.05895. 10.1080/01621459.2020.1769635 PMC9126075 35615339

[73] Anders S, Huber W (2010). Differential expression analysis for sequence count data. Genome Biol.

[74] Bullard JH, Purdom E, Hansen KD, Dudoit S (2010). Evaluation of statistical methods for normalization and differential expression in mRNA-seq experiments. BMC Bioinformatics.

[75] Robinson MD, Oshlack A (2010). A scaling normalization method for differential expression analysis of RNA-seq data. Genome Biol.

[76] Langmead B, Hansen KD, Leek JT (2010). Cloud-scale RNA-sequencing differential expression analysis with Myrna. Genome Biol.

[77] Dillies MA, Rau A, Aubert J, Hennequet-Antier C, Jeanmougin M, Servant N (2012). A comprehensive evaluation of normalization methods for Illumina high-throughput RNA sequencing data analysis. Brief Bioinformatics.

[78] Spearman C (1904). "General Intelligence," Objectively Determined and Measured. Am J Psychol.

[79] Hotelling H (1933). Analysis of a complex of statistical variables into principal components. J Educ Psychol.

[80] Eckart C, Young G (1936). The approximation of one matrix by another of lower rank. Psychometrika.

[81] Comon Pierre (1994). Independent component analysis, A new concept?. Signal Processing.

[82] Tipping ME, Bishop CM (1999). Probabilistic Principal Component Analysis. J R Stat Soc Ser B Stat Methodol.

[83] Lee DD, Seung HS (1999). Learning the parts of objects by non-negative matrix factorization. Nature.

[84] Hyvärinen A, Oja E (2000). Independent component analysis: algorithms and applications. Neural Netw.

[85] West M, Bernardo J, Bayarri M, Berger J, Dawid A, Heckerman D, Smith A (2003). Bayesian factor regression models in the “large *p*, small *n*" paradigm. Bayesian Statistics 7. Proceedings of the Seventh Valencia International Meeting.

[86] Zou H, Hastie T, Tibshirani R (2006). Sparse principal component analysis. J Comput Graph Stat.

[87] Hoff PD (2007). Model averaging and dimension selection for the singular value decomposition. J Amer Statist Assoc.

[88] Salakhutdinov R, Mnih A (2008). Bayesian Probabilistic Matrix Factorization Using Markov Chain Monte Carlo. Proceedings of the 25th International Conference on Machine Learning. ICML ’08.

[89] Ghosh J, Dunson DB (2009). Default prior distributions and efficient posterior computation in Bayesian factor analysis. J Comput Graph Stat.

[90] Witten DM, Tibshirani R, Hastie T (2009). A penalized matrix decomposition, with applications to sparse principal components and canonical correlation analysis. Biostatistics.

[91] Engelhardt BE, Stephens M (2010). Analysis of Population Structure: A Unifying Framework and Novel Methods Based on Sparse Factor Analysis. PLoS Genet.

[92] Mayrink VD, Lucas JE (2013). Sparse latent factor models with interactions: Analysis of gene expression data. Ann Appl Stat.

[93] Yang D, Ma Z, Buja A (2014). A Sparse Singular Value Decomposition Method for High-Dimensional Data. J Comput Graph Stat.

[94] Josse J, Wager S (2016). Bootstrap-Based Regularization for Low-Rank Matrix Estimation. J Mach Learn Res.

[95] Leung D, Drton M (2016). Order-invariant prior specification in Bayesian factor analysis. Stat Probab Lett.

[96] Wang W, Stephens M. Empirical Bayes Matrix Factorization. arXiv preprint arXiv:180206931. 2018. https://arxiv.org/abs/1802.06931.

[97] Buettner F, Natarajan KN, Casale FP, Proserpio V, Scialdone A, Theis FJ (2015). Computational analysis of cell-to-cell heterogeneity in single-cell RNA-sequencing data reveals hidden subpopulations of cells. Nat Biotechnol.

[98] Scialdone A, Natarajan KN, Saraiva LR, Proserpio V, Teichmann SA, Stegle O (2015). Computational assignment of cell-cycle stage from single-cell transcriptome data. Methods.

[99] Hansen BB, Klopfer SO (2006). Optimal Full Matching and Related Designs via Network Flows. J Comput Graph Stat.

[100] Gale D, Shapley LS (1962). College Admissions and the Stability of Marriage. Am Math Mon.

[101] Kuhn HW (1955). The Hungarian method for the assignment problem. Nav Res Logist Q.

[102] Zhang F, Horn RA. In: Zhang F, (ed).Basic properties of the Schur complement: Springer; 2005, pp. 17–46.

[103] Robinson MD, McCarthy DJ, Smyth GK (2009). edgeR: a Bioconductor package for differential expression analysis of digital gene expression data. Bioinformatics.

[104] Benjamini Yoav, Hochberg Yosef (1995). Controlling the False Discovery Rate: A Practical and Powerful Approach to Multiple Testing. Journal of the Royal Statistical Society: Series B (Methodological).

[105] Buja A, Eyuboglu N (1992). Remarks on parallel analysis. Multivar Behav Res.

[106] Zheng GX, Terry JM, Belgrader P, Ryvkin P, Bent ZW, Wilson R (2017). Massively parallel digital transcriptional profiling of single cells. Nat Commun.

[107] Risso D, Schwartz K, Sherlock G, Dudoit S (2011). GC-Content Normalization for RNA-Seq Data. BMC Bioinformatics.

[108] McInnes L, Healy J, Melville J. UMAP: Uniform Manifold Approximation and Projection for Dimension Reduction. Journal of Open Source Software. 2018; 3(29):861. The Open Journal. 10.21105/joss.00861. 10.21105/joss.00861.

[109] Maaten Lvd, Hinton G (2008). Visualizing data using t-SNE. J Mach Learn Res.

[110] Jonsson V, Österlund T, Nerman O, Kristiansson E (2016). Statistical evaluation of methods for identification of differentially abundant genes in comparative metagenomics. BMC genomics.

[111] The Genotype-Tissue Expression (GTEx) Project. GTEx Analysis V7. 2016. https://gtexportal.org. Accessed Jan 2020.

[112] Wickham H (2016). ggplot2: Elegant Graphics for Data Analysis.

[113] R Core Team. R: A Language and Environment for Statistical Computing. Vienna; 2019. https://www.R-project.org/.

