# Supplementary material for: Data-based RNA-seq simulations by binomial thinning
Source: BMC Bioinformatics. 2020 May 24;21:206. doi: 10.1186/s12859-020-3450-9 (PMC7245910; doi:10.1186/s12859-020-3450-9)
Supplement: Supplementary file 1 — Additional file 1 This PDF file contains theoretical considerations, simulation summaries and figures, and additional simulation details. [file 12859_2020_3450_MOESM1_ESM.pdf]

ADDITIONAL FILE 1

Supplement to: Data-based RNA-seq Simulations by Binomial Thinning

David Gerard

Correspondence:
dgerard@american.edu
Department of Mathematics and Statistics, American University, Massachusetts Ave NW, 20016 Washington, DC, USA
Full list of author information is available at the end of the article

Abstract

This document contains theoretical considerations, additional simulation details, and supplementary figures to "Data-based RNA-seq Simulations by Binomial Thinning."

S1 Theoretical Considerations

S1.1 Target Correlation

Theorem S1 *Let (u_i, z_i) be iid jointly standard normal with correlation ρ for $i = 1, 2, \ldots, n$. Let w_j be iid standard normal for $j = 1, 2, \ldots, n$. Suppose we match the w_j's onto the u_i's by order statistics, resulting in (w_i, u_i) pairs such that the rank of w_i is the same as the rank of u_i. Then* $\operatorname{cor}(w_i, z_i) \underset{n\to\infty}{\to} \rho$.

Proof For a fixed proportion p, we note that $u_{(\lceil np \rceil)}$ and $w_{(\lceil np \rceil)}$ converge in probability to the theoretical p-quantile of the standard normal distribution [1, e.g.]. Since the order statistics converge to the same values, and we match by order statistics, this implies that $u_i - w_i \xrightarrow{P} 0$. Thus, by Slutsky's theorem, we have that $u_i z_i - w_i z_i \xrightarrow{P} 0$.

We note that $|u_i z_i - w_i z_i| \leq |u_i z_i| + |w_i z_i|$, by the triangle inequality. The term on the right has finite expectation as (using Cauchy-Schwarz)

$$\mathrm{E}[|u_i z_i| + |w_i z_i|] \leq \mathrm{E}[u_i^2]^{1/2}\,\mathrm{E}[z_i^2]^{1/2} + \mathrm{E}[w_i^2]^{1/2}\,\mathrm{E}[z_i^2]^{1/2} = 2. \tag{S1}$$

Thus, by the Lebesgue dominated convergence theorem, we have $\mathrm{E}[|u_i z_i - w_i z_i|] \to 0$. Since $-|u_i z_i - w_i z_i| \leq u_i z_i - w_i z_i \leq |u_i z_i - w_i z_i|$, this implies that $\mathrm{E}[u_i z_i] - E[w_i z_i] \to 0$, and the theorem is proved.

□

To place the results of Theorem S1 in context of the matching in Procedure 2, note that the u_i's are the elements of $\boldsymbol{U}$, the z_i's are the elements of $\hat{\boldsymbol{Z}}$, the w_i's are the elements of $\boldsymbol{X}_3$, ρ is the target correlation between the one column in $\boldsymbol{X}_3$ and the one column in $\hat{\boldsymbol{Z}}$, and $\boldsymbol{\Pi}$ is the permutation matrix that results in the matching of the w_i's and the u_i's.

The results of Theorem S1 can be generalized to non-standard normal distributions by appealing to the weak law of large numbers and Slutsky's theorem.

S1.2 Generalizing the Poisson Assumption

For simplicity, we stated a Poisson distribution as the modeling assumption in (1). However, our methods are equally valid under more general conditions. We begin by showing how our methods are valid when using the negative binomial distribution, which is perhaps the most common distribution used to analyze RNA-seq counts [2–4]. To see this, we prove the following simple lemma which, though less well-known than Lemma 1, can still be found in some elementary texts (or at least a version of the following lemma) [exercise 4.32 of 5, e.g.].

Lemma S1 *Suppose* $y \sim \text{NB}(\mu, \phi)$*, where we are using the parameterization such that* $\text{E}[y] = \mu$ *and* $\text{var}(y) = \mu(1 + \mu\phi)$*. Also suppose that* $\tilde{y}|y \sim \text{Bin}(y, p)$*. Then* $\tilde{y} \sim \text{NB}(p\mu, \phi)$*.*

Proof Using the hierarchical characterization of the negative binomial distribution, we have that

$$\lambda \sim \text{Gamma}(1/\phi, \mu\phi) \quad \text{(S2)}$$

$$y|\lambda \sim \text{Poisson}(\lambda), \quad \text{(S3)}$$

where $1/\phi$ is the shape parameter and $\mu\phi$ is the scale parameter. This implies that $\tilde{y}|\lambda \sim \text{Poisson}(p\lambda)$. But $p\lambda \sim \text{Gamma}(1/\phi, p\mu\phi)$ by elementary properties of the gamma distribution. Hence, by the hierarchical characterization of the negative binomial distribution, we have that $\tilde{y} \sim \text{NB}(p\mu, \phi)$. □

The zero-inflated negative binomial distribution is sometimes used to model single-cell RNA-seq data as it can account for the abundance of zeros observed in such data [6–8]. A random variable y is distributed zero-inflated negative binomial, denoted $y \sim \text{ZINB}(\pi, \mu, \phi)$, if it is generated by the following hierarchical process:

$$z \sim \text{Bern}(1 - \pi), \quad \text{(S4)}$$

$$y|z = 0 \sim \delta_0 \quad \text{(S5)}$$

$$y|z = 1 \sim \text{NB}(\mu, \phi), \quad \text{(S6)}$$

where δ_0 is the degenerate distribution with a point-mass at 0. In words, the counts are either 0 with probability π or follow a negative binomial distribution with probability $1 - \pi$. Our methods are equally valid in the zero-inflated negative binomial case.

Lemma S2 *Suppose* $y \sim \text{ZINB}(\pi, \mu, \phi)$ *and* $\tilde{y}|y \sim \text{Bin}(y, p)$*. Then* $\tilde{y} \sim \text{ZINB}(\pi, p\mu, \phi)$*.*

Proof It's sufficient to note that

$$\tilde{y}|z = 0 \sim \delta_0, \text{ and} \quad \text{(S7)}$$

$$\tilde{y}|z = 1 \sim \text{NB}(p\mu, \phi). \quad \text{(S8)}$$

□

Finally, our simulation methods preserve the count distribution in the rich class of distributions which are mixtures of binomial and negative binomial distributions (some examples within this class of distributions are plotted in Supplementary Figure S1).

Lemma S3 *Let $\pi_0, \pi_1, \ldots \pi_M$ and $\tau_1, \tau_2, \ldots, \tau_L$ be non-negative mixing proportions such that*

$$\sum_{m=0}^{M} \pi_m + \sum_{\ell=1}^{L} \tau_\ell = 1. \tag{S9}$$

Suppose that y has a PMF which is a mixture of binomial and negative binomial PMF's

$$f(y) = \pi_0 \delta_0(y) + \sum_{m=1}^{M} \pi_m \,\mathrm{NB}(y|\mu_m, \phi_m) + \sum_{\ell=1}^{L} \tau_\ell \,\mathrm{Bin}(y|\frac{\nu_\ell}{n_\ell}, n_\ell), \tag{S10}$$

where $\mathrm{NB}(y|\mu_m, \phi_m)$ is the negative binomial PMF with mean μ_m and dispersion ϕ_m, and $\mathrm{Bin}(y|\frac{\nu_\ell}{n_\ell}, n_\ell)$ is the binomial PMF with mean ν_ℓ and success probability ν_ℓ/n_ℓ. Suppose that $\tilde{y}|y \sim \mathrm{Bin}(y, p)$. Then

$$\mathrm{E}[\tilde{y}] = p\,\mathrm{E}[y], \text{ and} \tag{S11}$$

$$f(\tilde{y}) = \pi_0 \delta_0(\tilde{y}) + \sum_{m=1}^{M} \pi_m \,\mathrm{NB}(\tilde{y}|p\mu_m, \phi_m) + \sum_{\ell=1}^{L} \tau_\ell \,\mathrm{Bin}(\tilde{y}|\frac{p\nu_\ell}{n_\ell}, n_\ell). \tag{S12}$$

Proof Equation (S11) is just a consequence of the law of total expectation. To prove (S12), note that if $y \sim \mathrm{NB}(\mu_m, \phi_m)$ then $\tilde{y} \sim \mathrm{NB}(p\mu_m, \phi_m)$ and if $y \sim \mathrm{Bin}(\nu_\ell/n_\ell, n_\ell)$ then $\tilde{y} \sim \mathrm{Bin}(p\nu_\ell/n_\ell, n_\ell)$. The proof follows by conditioning on the latent mixing group. □

S2 Additional Simulations

S2.1 Correlation Estimator

We explored the effects of changing the target correlation on the true correlation. We varied the sample size, $N \in \{6, 10, 20, 500\}$, and the target correlations between $\boldsymbol{z}$ and the two columns in $\boldsymbol{\Pi X}_3$, $\boldsymbol{r} \in \{(0,0), (0.5,0), (0.9,0), (0.5,0.5)\}$. We did not implement simulations for $\boldsymbol{r} = (0.5, 0.9)$ because the resulting correlation matrix would not be positive definite. Under each unique combination of simulation parameter settings, we iterative drew $\boldsymbol{z} \in \mathbb{R}^N$ from a standard normal. We also drew $\boldsymbol{X}_3 \in \mathbb{R}^{N\times 2}$ according to two schemes:

1. Normal: Each element of $\boldsymbol{X}_3$ is independently drawn from a standard normal distribution, and
2. Indicator: The first column of $\boldsymbol{X}_3$ consists of $(1, 0, 1, 0, \ldots, 1, 0)^\intercal$, and the second column of $\boldsymbol{X}_3$ consists of $(\boldsymbol{1}_{N/2}^\intercal, \boldsymbol{0}_{N/2}^\intercal)^\intercal$.

Each replicate, we used Procedure 4 to estimate the correlation between $\boldsymbol{\Pi X}_3$ and $\boldsymbol{z}$. We did this for a total of 100 replications for each combination of simulation parameters.

The results are presented in Supplementary Figure S15. Because we are approximating the expected (conditional on $\boldsymbol{z}$) Pearson correlation between the columns of $\boldsymbol{\Pi X}_3$ and $\boldsymbol{z}$, the true correlations between $\boldsymbol{\Pi X}_3$ and $\boldsymbol{z}$ are approximately the mean of the estimates over the 100 replications (see (17)). From Supplementary Figure S15, we note that the true correlation is generally closer to 0 than the target correlation. When the sample size is 20, there seems to be very little variability in the correlation estimates.

S2.2 Single Cell Factor Analysis Simulations

In this section, we evaluate the same five factor analysis methods from Section 3.3 using a single cell RNA-seq dataset. We used a dataset of Peripheral Blood Mononuclear Cells (PBMC) from 10X Genomics [9], providing the same filters as the tutorial for Seurat [10, 11], available at `https://satijalab.org/seurat/v3.1/pbmc3k_tutorial.html`. Specifically, we removed cells that have unique feature counts either over 2500 or less than 200, and we removed cells that have more than 5% mitochondrial counts. After filtering cells, we kept the 4000 features with the greatest cell-to-cell variation in the dataset, where variation was measured after normalizing each cell. This resulted in a dataset of 4000 features on 2638 cells.

To simulate single-cell RNA-seq data, we took the filtered PBMC data, assumed model (9), then generated data using `seqgendiff` such that (19) holds. We simulated the components of $\boldsymbol{x}_3$ and the non-zero components of $\boldsymbol{b}_3$ from independent normal distributions. We varied the following parameters of the simulation study:

1 The signal strength: the standard deviation of the loadings (the b_{3g}'s) was set to one of $\{0.4, 0.8\}$, with higher standard deviations corresponding to higher signal.

2 The sparsity: the proportion of loadings (the b_{3g}'s) that are 0 was set to one of $\{0, 0.9\}$, and

3 The target correlations of the added factor with the first unobserved factor: $r \in \{0, 0.5\}$.

We fixed the number of genes to be 1000 and the number of cells to be 500. Thus, we had eight different simulation settings under evaluation. We ran 100 replications for each simulation setting, each time fitting one of the five factor analysis methods. We applied all factor analysis methods to the $\log_2$-counts, after adding half a pseudocount. The number of factors was estimated using parallel analysis [12].

We used the same three metrics to evaluate the different factor analysis methods as in Section 3.3. The results are presented in Figures S18, S19, and S20. We have the following conclusions:

1 PEER generally performed poorly, only excelling in estimating the loadings in high-signal and low-sparsity regimes.

2 ICA had difficulty estimating the factors in high sparsity, high correlation regimes.

3 PCA, SSVD, and *flash* all performed comparably.

Based on these conclusions, we would suggest researchers explore PCA, SSVD, or *flash*.

S2.3 SimSeq Simulations

We ran the SimSeq simulation software [13] using the GTEx muscle data [14]. SimSeq requires the presence of a group indicator variable to create differentially expressed genes. So we used the individual's sex as this indicator variable. We simulated data so that 90% of genes are non-differentially expressed, using 10000 genes and a sample of size 10 individuals each replication (5 for each group). For each of 500 repetitions, we calculated the false positive rate and the power when using DESeq2 [4], edgeR [15], and voom-limma [16].

Since the performance of SimSeq depends on the quality of the indicator variable used, we estimated the proportion of non-null genes between sexes using voom-limma-eBayes [16] and *ash* [17]. *Ash* estimated that 98.2% of genes are differentially expressed, indicating that sex is an appropriate indicator variable to use for SimSeq. Many of these observed associations are likely due to unwanted variation [18]. But for the purposes of the SimSeq simulation method, it is only important to have an indicator variable that is marginally associated with gene expression.

SimSeq is unable to control the strength of the signal between two groups. So for accurate comparison with seqgendiff, we needed to estimate the distribution of effect sizes. We used *ash* to estimate the standard deviation of effect sizes in the GTEx muscle data, resulting in an estimate of 0.334. We then ran seqgendiff using a sample size of 10 (5 for each of two groups), 10000 genes, setting 90% of the genes to be null, and drawing the effect sizes of the non-null genes from a normal distribution with mean 0 and standard deviation 0.334. We did this for 500 repetitions, each time calculating the false positive rate and power when using DESeq2, edgeR, and voom-limma.

The results are presented in Figure S16. There, we see that false positive rates and powers are distributionally similar when using either seqgendiff or SimSeq. Thus, when seqgendiff uses effect sizes approximately similar to the indicator variable that SimSeq uses, the results are about the same.

References

1. Arnold BC, Balakrishnan N, Nagaraja HN. A first course in order statistics. vol. 54. Siam; 1992.
2. Robinson MD, Smyth GK. Moderated statistical tests for assessing differences in tag abundance. Bioinformatics. 2007 09;23(21):2881–2887.
3. Robinson MD, Smyth GK. Small-sample estimation of negative binomial dispersion, with applications to SAGE data. Biostatistics. 2007 08;9(2):321–332.
4. Love MI, Huber W, Anders S. Moderated estimation of fold change and dispersion for RNA-seq data with DESeq2. Genome Biology. 2014 Dec;15(12):550.
5. Casella G, Berger RL. Statistical inference. vol. 2. Duxbury Pacific Grove, CA; 2002.
6. Miao Z, Deng K, Wang X, Zhang X. DEsingle for detecting three types of differential expression in single-cell RNA-seq data. Bioinformatics. 2018 04;34(18):3223–3224.
7. Risso D, Perraudeau F, Gribkova S, Dudoit S, Vert JP. A general and flexible method for signal extraction from single-cell RNA-seq data. Nature communications. 2018;9(1):284.
8. Eraslan G, Simon LM, Mircea M, Mueller NS, Theis FJ. Single-cell RNA-seq denoising using a deep count autoencoder. Nature communications. 2019;10(1):390.
9. Zheng GX, Terry JM, Belgrader P, Ryvkin P, Bent ZW, Wilson R, et al. Massively parallel digital transcriptional profiling of single cells. Nature communications. 2017;8:14049.
10. Butler A, Hoffman P, Smibert P, Papalexi E, Satija R. Integrating single-cell transcriptomic data across different conditions, technologies, and species. Nature biotechnology. 2018;36(5):411.
11. Stuart T, Butler A, Hoffman P, Hafemeister C, Papalexi E, Mauck III WM, et al. Comprehensive Integration of Single-Cell Data. Cell. 2019;.
12. Buja A, Eyuboglu N. Remarks on parallel analysis. Multivariate behavioral research. 1992;27(4):509–540.
13. Benidt S, Nettleton D. SimSeq: a nonparametric approach to simulation of RNA-sequence datasets. Bioinformatics. 2015 02;31(13):2131–2140.
14. GTEx Consortium. Genetic effects on gene expression across human tissues. Nature. 2017;550(7675):204.
15. Robinson MD, McCarthy DJ, Smyth GK. edgeR: a Bioconductor package for differential expression analysis of digital gene expression data. Bioinformatics. 2009 11;26(1):139–140.

16. Law CW, Chen Y, Shi W, Smyth GK. Voom: precision weights unlock linear model analysis tools for RNA-seq read counts. Genome biology. 2014;15(R29).
17. Stephens M. False discovery rates: a new deal. Biostatistics. 2016 10;18(2):275–294.
18. Leek JT, Scharpf RB, Bravo HC, Simcha D, Langmead B, Johnson WE, et al. Tackling the widespread and critical impact of batch effects in high-throughput data. Nature Reviews Genetics. 2010;11(10):733–739.
19. Smyth GK. Linear models and empirical Bayes methods for assessing differential expression in microarray experiments. Statistical Applications in Genetics and Molecular Biology. 2004;3(1).

Supplementary Figures

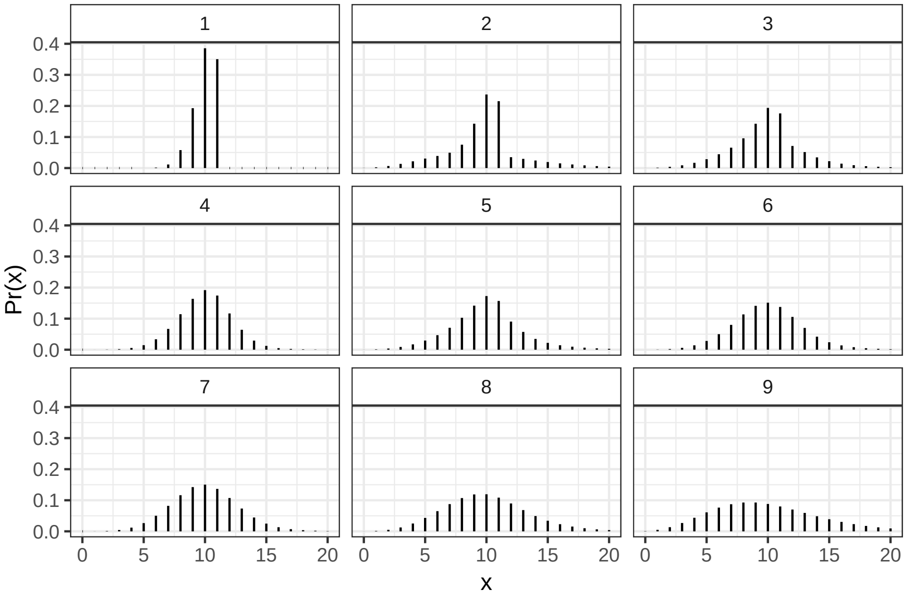

Figure S1 Some distributions in the class of mixtures of binomial and negative binomial distributions, demonstrating the flexibility of this class. The mean was set to 10 for all mixing components.

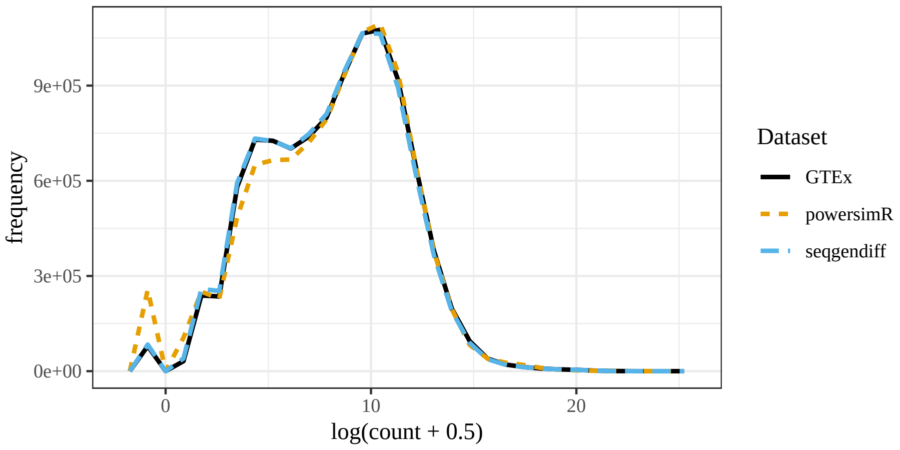

Figure S2 Frequency polygons for the $\log_2$ counts for the dataset simulated by `seqgendiff` (blue), the dataset simulated by `powsimR` (orange), and the GTEx muscle dataset (black). The `seqgendiff` and GTEx count distributions are almost exactly the same.

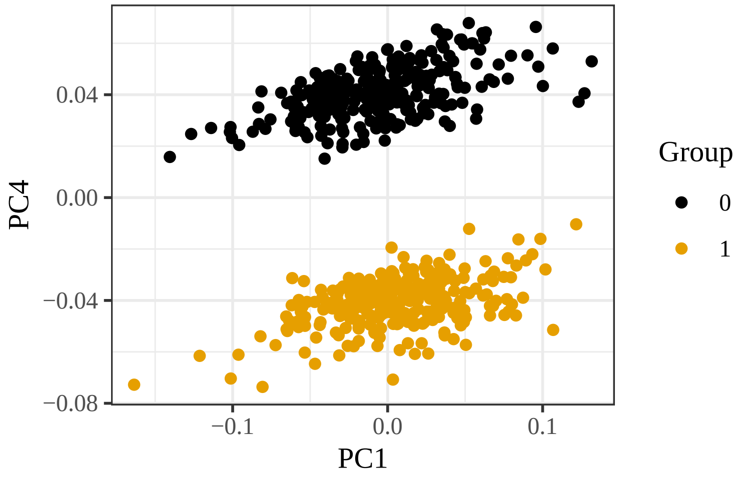

Figure S3 First principle component (x-axis) versus the fourth principle component (y-axis) in the `seqgendiff` dataset. The fourth principle component seems capture group membership.

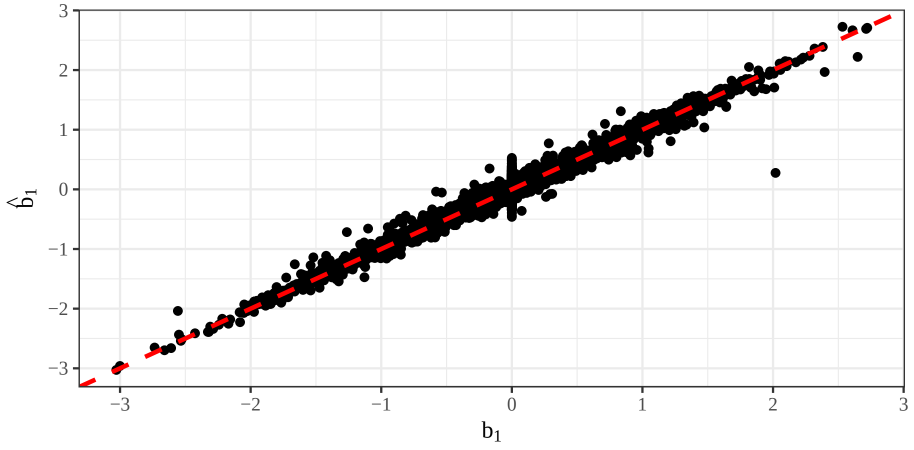

Figure S4 True coefficient values (x-axis) versus their corresponding estimates (y-axis) in the `seqgendiff` dataset. Estimates were obtained using the voom-limma pipeline [16, 19].

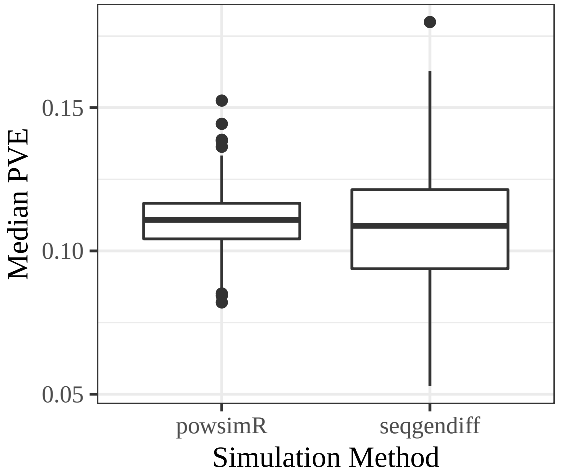

Figure S5 Median (across non-null genes) proportion of variance explained (18) (y-axis) for datasets generated by `powsimR` or `seqgendiff` (x-axis). The two sets of simulated datasets have the same expected median PVE, though the median PVE is more variable among the `seqgendiff` datasets.

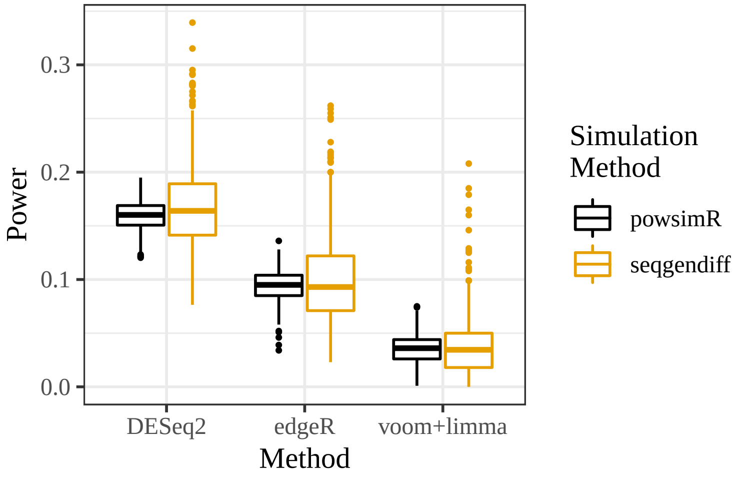

Figure S6 Boxplots of power (y-axis) for various differential expression analysis methods (x-axis) when applied on different simulated datasets (color).

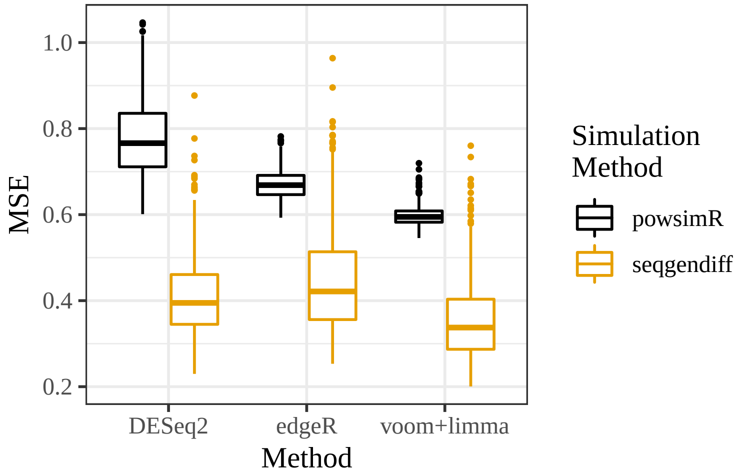

Figure S7 Boxplots of mean squared error (y-axis) for various differential expression analysis methods (x-axis) when applied on different simulated datasets (color).

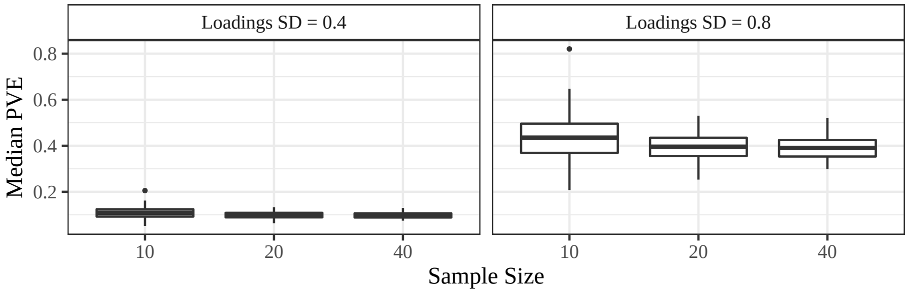

Figure S8 Median proportion of variance explained (18) for the genes with non-zero loadings (y-axis) stratified by the sample size (x-axis) from the simulation study in Section 3.3. The facets index the different standard deviations of the loadings.

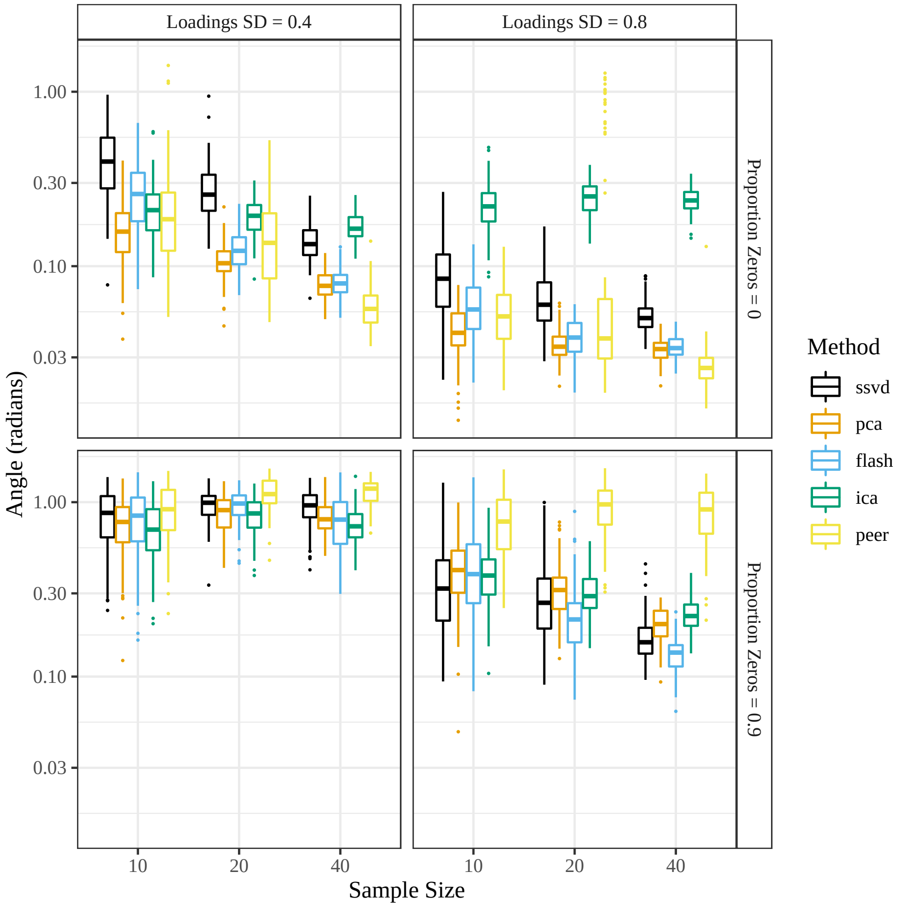

Figure S9 Angle ($\log_{10}$-scale) between the added factor and its projection onto the column space of the estimated factors (y-axis), stratified by sample size (x-axis), factor analysis method (color), signal strength (column facets), and sparsity of the loadings (row facets). The target correlation between the added factor and the first unobserved factor was set to 0. A smaller angle is better.

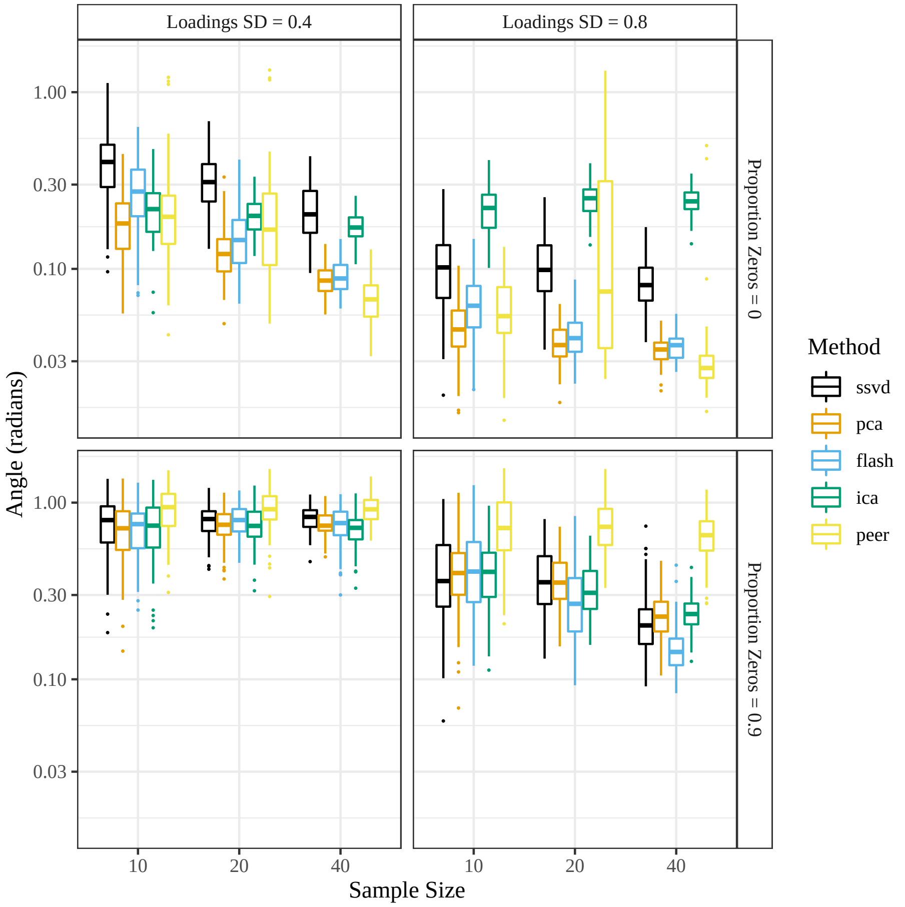

Figure S10 Angle ($\log_{10}$-scale) between the added factor and its projection onto the column space of the estimated factors (y-axis), stratified by sample size (x-axis), factor analysis method (color), signal strength (column facets), and sparsity of the loadings (row facets). The target correlation between the added factor and the first unobserved factor was set to 0.5. A smaller angle is better.

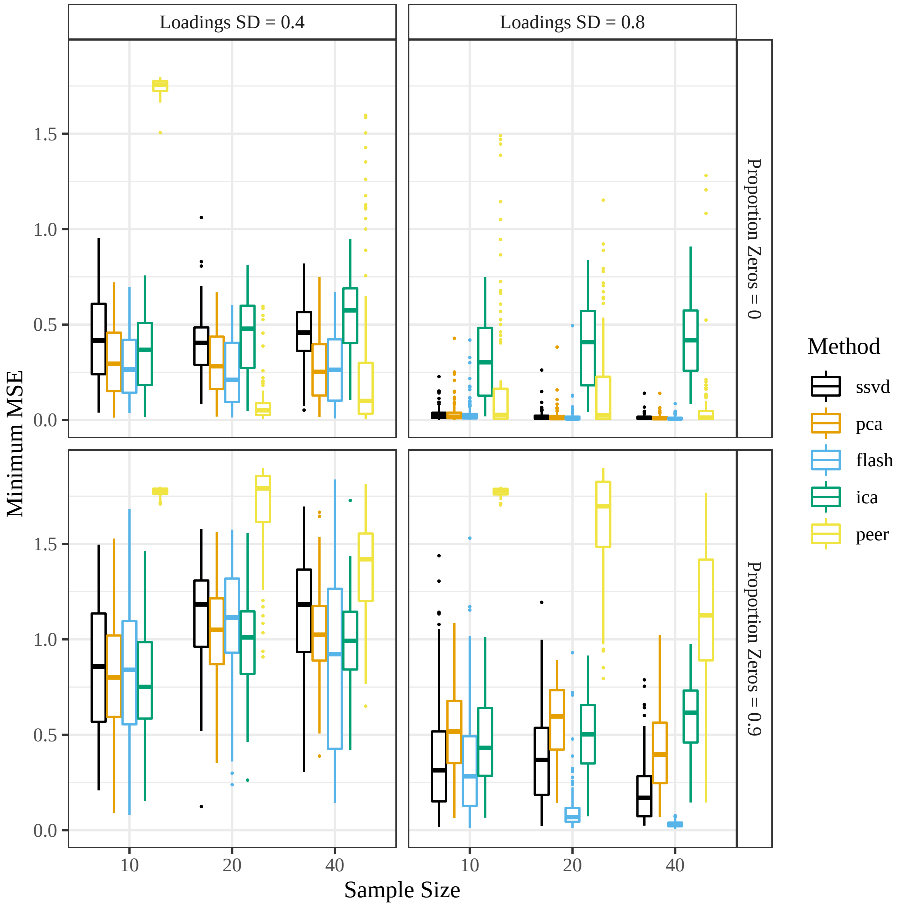

Figure S11 Minimum mean squared error between the added factor and the estimated factors (y-axis), stratified by sample size (x-axis), factor analysis method (color), signal strength (column facets), and of the loadings sparsity (row facets). Before calculating the MSE, all factors were scaled to have ℓ^2-norm of 1. The target correlation between the added factor and the first unobserved factor was set to 0. A smaller minimum MSE is better.

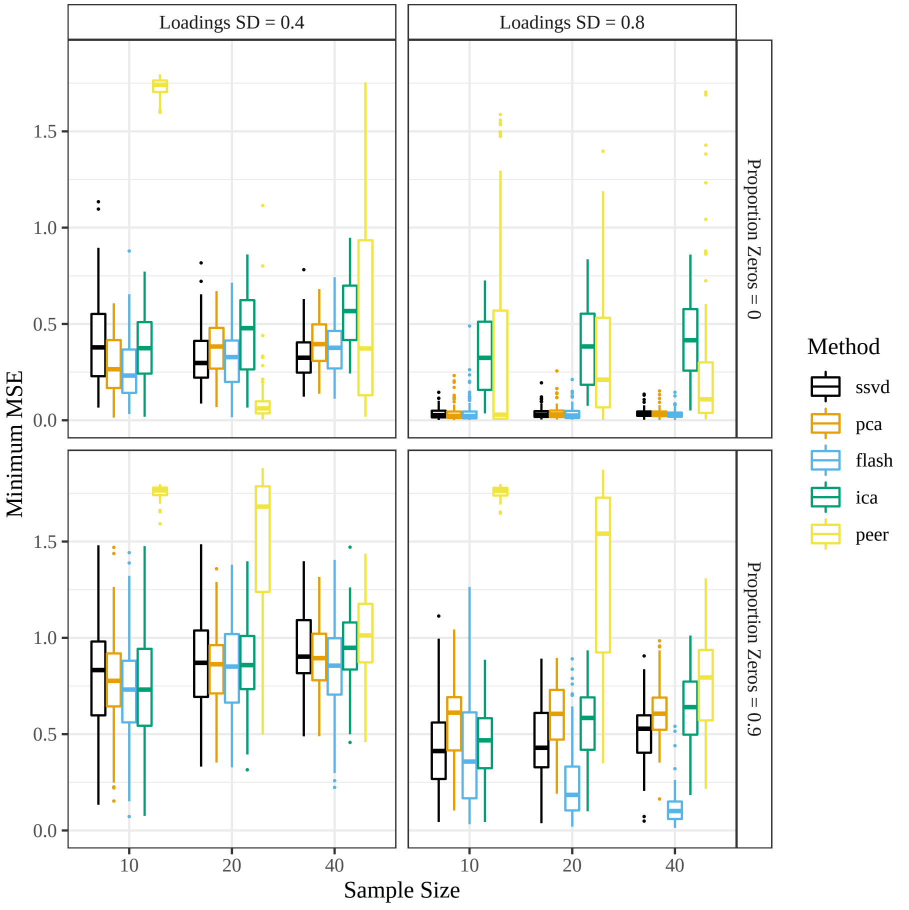

Figure S12 Minimum mean squared error between the added factor and the estimated factors (y-axis), stratified by sample size (x-axis), factor analysis method (color), signal strength (column facets), and sparsity of the loadings (row facets). Before calculating the MSE, all factors were scaled to have ℓ^2-norm of 1. The target correlation between the added factor and the first unobserved factor was set to 0.5. A smaller minimum MSE is better.

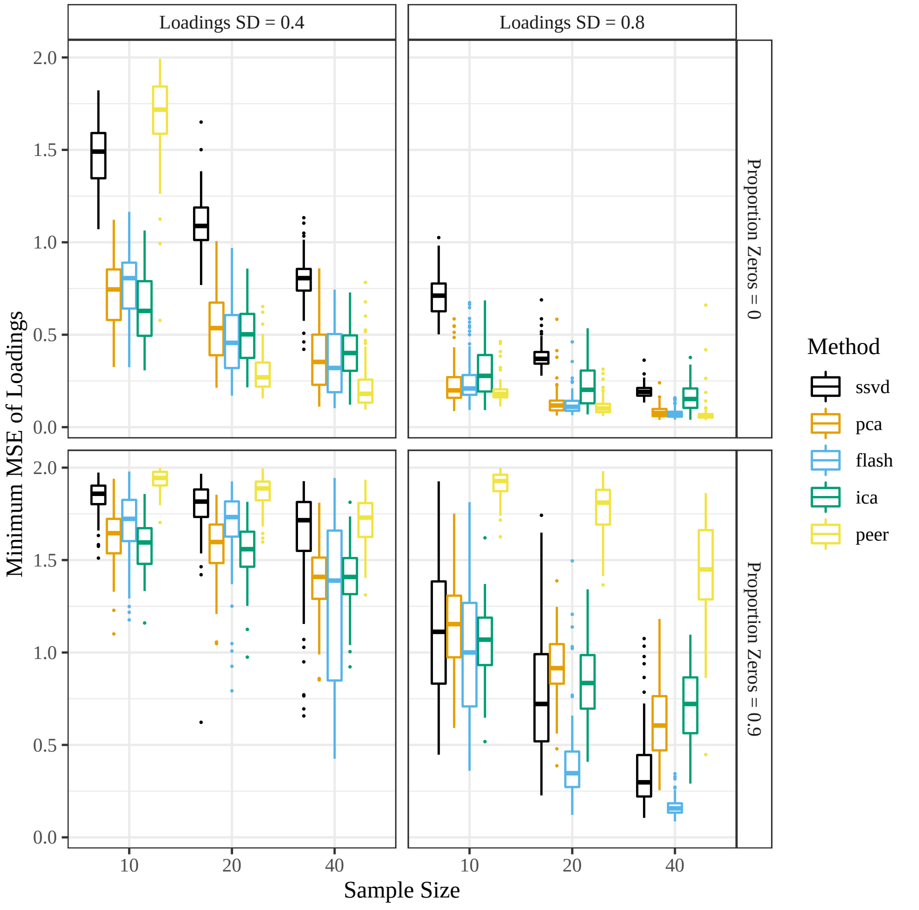

Figure S13 Minimum mean squared error between the added loading and the estimated loadings (y-axis), stratified by the sample size (x-axis), the factor analysis method (color), signal strength (column facets), and sparsity of the loadings (row facets). Before calculating the MSE, all loadings were scaled to have ℓ^2-norm of 1. The target correlation between the added factor and the first unobserved factor was set to 0. A smaller minimum MSE is better.

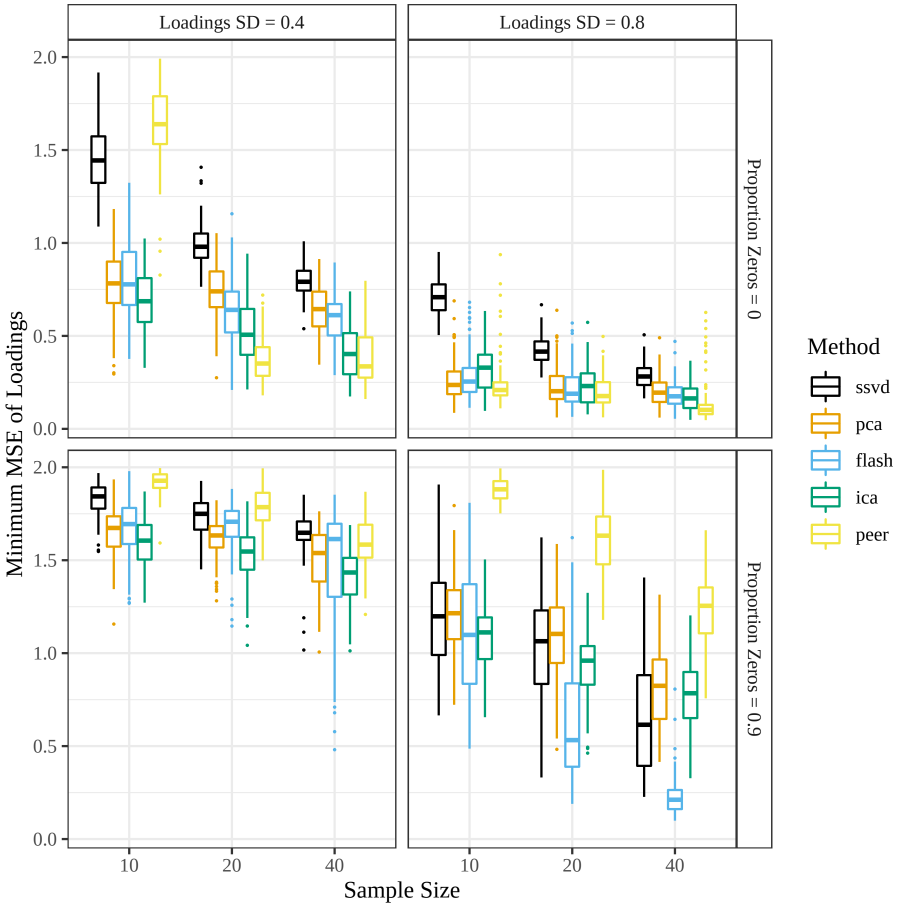

Figure S14 Minimum mean squared error between the added loading and the estimated loadings (y-axis), stratified by the sample size (x-axis), the factor analysis method (color), signal strength (column facets), and sparsity of the loadings (row facets). Before calculating the MSE, all loadings were scaled to have ℓ^2-norm of 1. The target correlation between the added factor and the first unobserved factor was set to 0.5. A smaller minimum MSE is better.

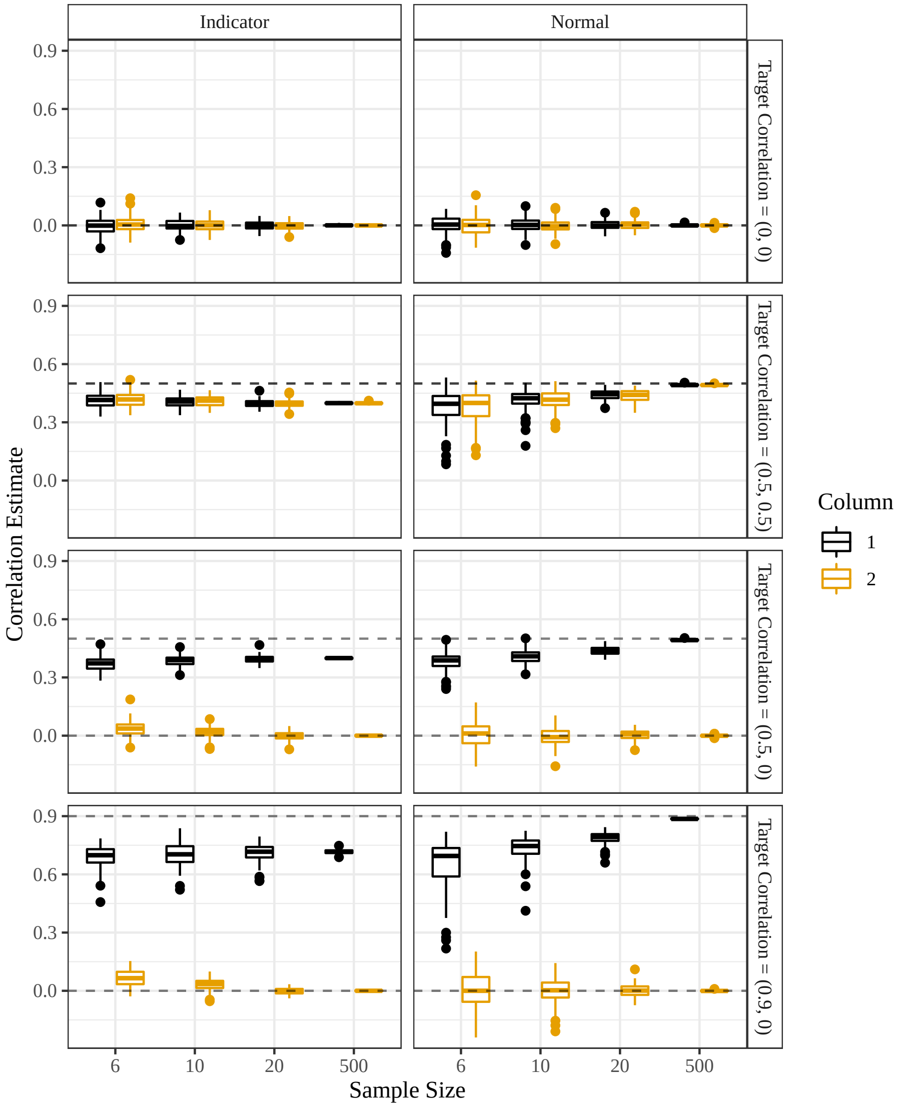

Figure S15 Boxplots of the Monte Carlo correlation estimator from Procedure 4 (y-axis), stratified by sample size (x-axis), the type of design matrix (column facets), the target correlations (row facets), and the column of the design matrix (color). Horizontal dashed lines are the two target correlations. The mean of the correlation estimates is approximately the true correlation.

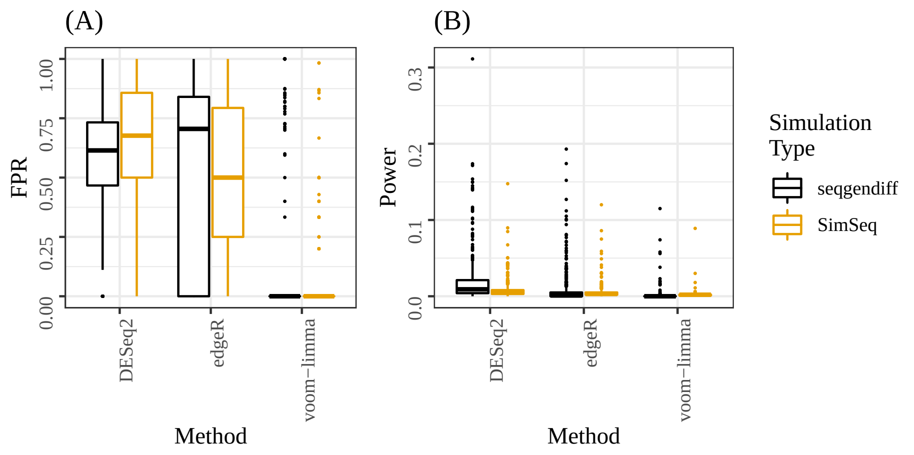

Figure S16 Boxplots of false positive rate (A) or power (B) stratified by differential expression analysis method (x-axis) and color-coded by data-based simulation technique (either `seqgendiff` or `SimSeq`) using the GTEx data [14]. Results are similar for each data-based simulation technique.

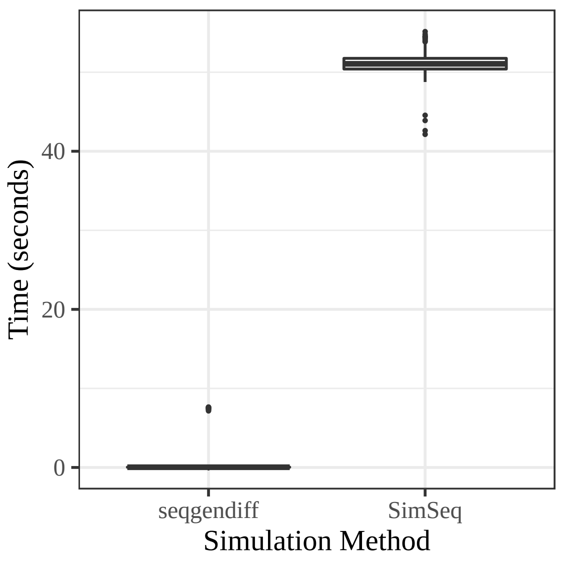

Figure S17 Boxplots of simulation time (y-axis) stratified by data-based simulation technique (x-axis) for the simulations run in Section S2.3. `SimSeq` is much slower than `seqgendiff`.

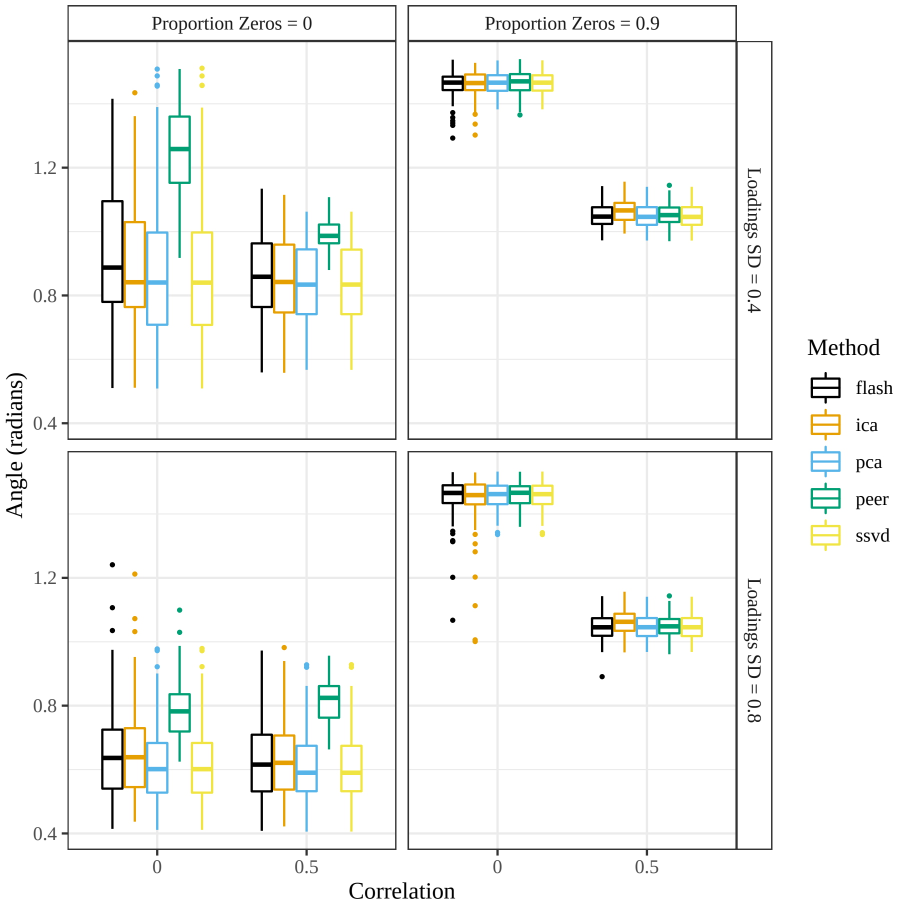

Figure S18 Angle between the added factor and its projection onto the column space of the estimated factors (y-axis), stratified by target correlation(x-axis), factor analysis method (color), signal strength (row facets), and sparsity of the loadings (column facets). A smaller angle is better.

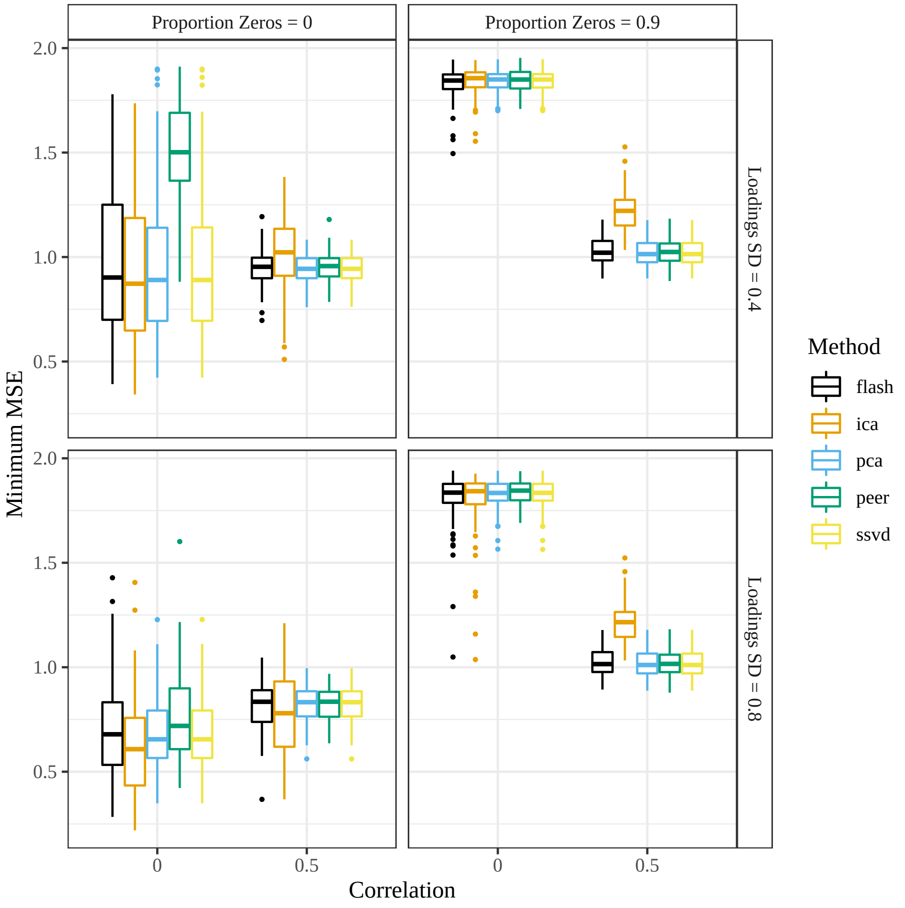

Figure S19 Minimum mean squared error between the added factor and the estimated factors (y-axis) on the single cell dataset, stratified by target correlation (x-axis), factor analysis method (color), signal strength (row facets), and sparsity of the loadings (column facets). Before calculating the MSE, all factors were scaled to have ℓ^2-norm of 1. A smaller minimum MSE is better.

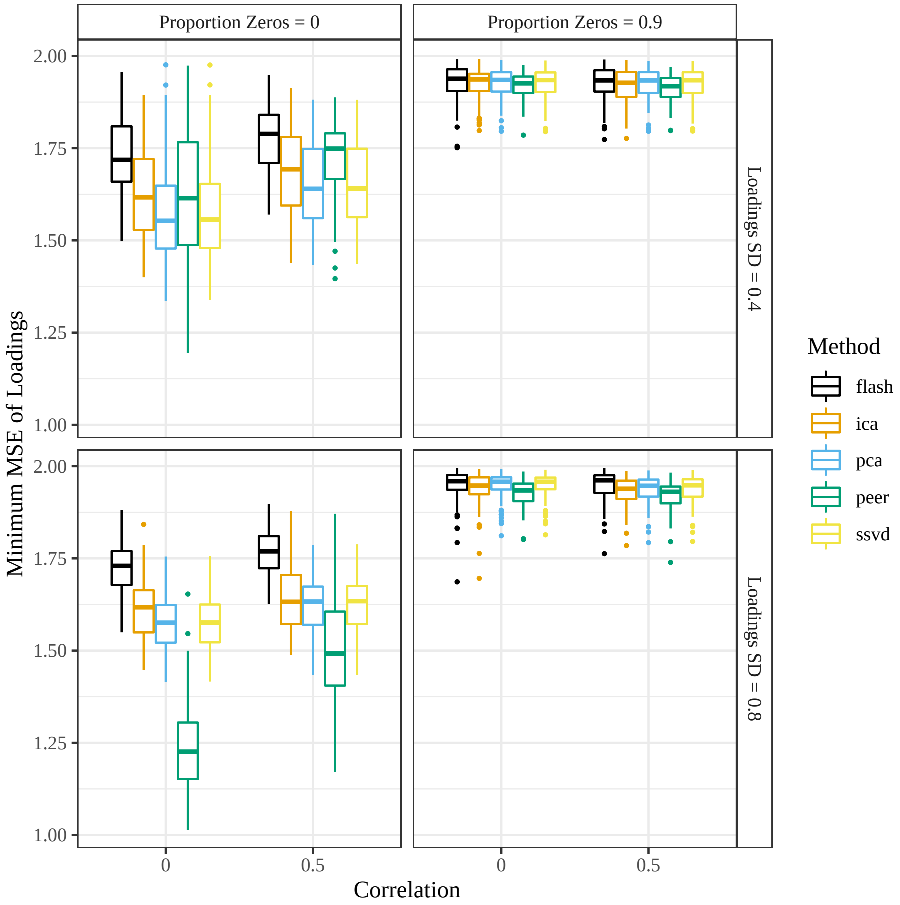

Figure S20 Minimum mean squared error between the added loading and the estimated loadings (y-axis) on the single cell dataset, stratified by target correlation (x-axis), factor analysis method (color), signal strength (row facets), and sparsity of the loadings (column facets). Before calculating the MSE, all factors were scaled to have ℓ^2-norm of 1. A smaller minimum MSE is better.
